# Utility of optical see-through head mounted displays in augmented reality-assisted surgery: A systematic review

**DOI:** 10.1016/j.media.2022.102361

**Published:** 2022-04

**Authors:** Manuel Birlo, P.J. Eddie Edwards, Matthew Clarkson, Danail Stoyanov

**Affiliations:** Wellcome/EPSRC Centre for Interventional and Surgical Sciences (WEISS), University College London (UCL), Charles Bell House, 43-45 Foley Street, London W1W 7TS, UK

**Keywords:** Augmented reality, Head-mounted displays, Optical see-through, Human factors

## Abstract

This article presents a systematic review of optical see-through head mounted display (OST-HMD) usage in augmented reality (AR) surgery applications from 2013 to 2020. Articles were categorised by: OST-HMD device, surgical speciality, surgical application context, visualisation content, experimental design and evaluation, accuracy and human factors of human-computer interaction. 91 articles fulfilled all inclusion criteria. Some clear trends emerge. The Microsoft HoloLens increasingly dominates the field, with orthopaedic surgery being the most popular application (28.6%). By far the most common surgical context is surgical guidance (n=58) and segmented preoperative models dominate visualisation (n=40). Experiments mainly involve phantoms (n=43) or system setup (n=21), with patient case studies ranking third (n=19), reflecting the comparative infancy of the field. Experiments cover issues from registration to perception with very different accuracy results. Human factors emerge as significant to OST-HMD utility. Some factors are addressed by the systems proposed, such as attention shift away from the surgical site and mental mapping of 2D images to 3D patient anatomy. Other persistent human factors remain or are caused by OST-HMD solutions, including ease of use, comfort and spatial perception issues. The significant upward trend in published articles is clear, but such devices are not yet established in the operating room and clinical studies showing benefit are lacking. A focused effort addressing technical registration and perceptual factors in the lab coupled with design that incorporates human factors considerations to solve clear clinical problems should ensure that the significant current research efforts will succeed.

## Highlights

• We present a systematic review of optical see-through head mounted display (OST-HMD) usage in augmented reality surgery applications from 2013 to 2020.
• 91 articles that fulfilled all inclusion criteria were categorized by OST- HMD device, surgical speciality, surgical application context, visualisation content, experimental design and evaluation, accuracy and human factors of human-computer interaction.
• Human Factors emerge as significant to OST-HMD utility.
• The significant upward trend in published articles is clear, but such devices are not yet established in the operating room and clinical studies showing benefit are lacking.
• A focused effort addressing technical registration and perceptual factors in the lab coupled with design that incorporates human factors considerations to solve clear clinical problems should ensure that the significant current research efforts will succeed.

## Graphical abstract

**Figure:**
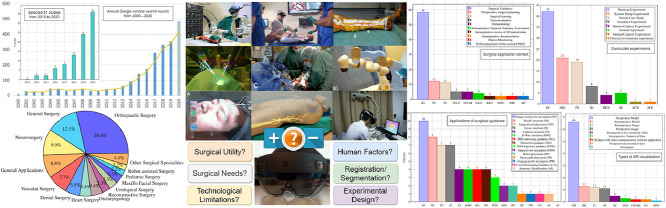

## 1 Introduction

**Fig. 1:**
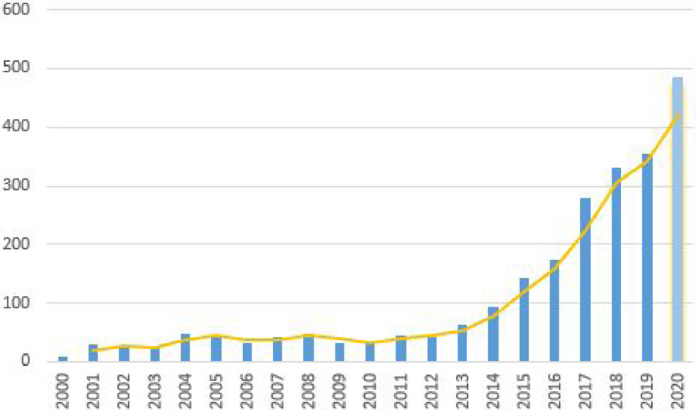
Google Scholar search results for surgery “Head Mounted Display” “Augmented Reality” OR “Mixed Reality” surgery “Head Mounted Display” “Augmented Reality” OR “Mixed Reality” “optical see through” OR “Hololens” OR “Magic Leap” OR “Google Glass” in the last 20 years (2020 results have a more recent search date)

Augmented reality (AR) surgical guidance was originally proposed in neurosurgery over 35 years ago (Kelly, Alker George, Goerss, 1982, Roberts, Strohbehn, Hatch, Murray, Kettenberger, 1986). The ability to view patient models directly on the surgeon’s view promises numerous benefits, including better perception, ergonomics, hand-eye coordination, safety, reliability, repeatability, and ultimately improved surgical outcomes. But despite more than three decades of research, the promise of AR has not yet translated into routine clinical practice. The development of commercial optical see-through head-mounted displays (OST-HMDs) including Google Glass, Moverio and the HoloLens have led to an increasing interest in such devices for surgical guidance (see Fig. 1). While there is occasional critical analysis (Carbone et al., 2020), the majority of authors tend to emphasise the great potential of AR in surgical applications. Multiple review papers for different specialities follow a similar pattern of positivity, but note that further research is needed. The lack of research demonstrating clinical benefit has been widely noted.

There is a risk that enthusiasm for the newly available OST-HMD devices may lead research along a similar path to earlier work and fail to achieve translation into routine surgery. The purpose of this paper is to provide a summary of current research in OST-AR in surgery and examine possible barriers to clinical adoption of such devices. We categorise papers according to application area, consider registration and validation methodologies as well as choice of visual content. Human factors emerge as a significant set of issues potentially limiting the applicability of AR and we provide a description of the most common issues encountered.

We believe that clinically successful AR can be achieved by clear identification of the critical points in specific surgical procedures where guidance is needed, identification of the relevant information segmented from preoperative scans and attention to human factors issues regarding the modes of visualisation and interaction. We hope that this review will be useful to clinicians and engineers collaborating on new OST-HMD AR projects and recommend consideration of human factors at an early stage.

## 2 Background

AR systems can be realised using different types of display media such as conventional displays, projectors or head-mounted displays (HMDs) (Sielhorst, Feuerstein, Navab, 2008, Okamoto, Onda, Yanaga, Suzuki, Hattori, 2015). Of these three display media categories, HMDs offer the most user-friendly solution for manual tasks since the user can work both from a self-centered perspective and hands-free (Condino et al., 2020). HMDs can be classified according to their underlying AR paradigm: Video see-through (VST) or optical see-through (OST).

In VST systems, a video image feed is combined with superimposed computer generated images such as 3D reconstructed organs. VST systems have been adopted in surgical applications via computer displays and HMDs, and have several potential advantages including improved synchronisation between video feed and overlay as well as video processing for image segmentation or registration. Also the contrast between video feed and overlaid graphical content can be easily controlled and the real scene can be occluded by a virtual overlay. The disadvantages of VST systems include limitations in terms of video bandwidth, the risk of losing vision of the real scene in the case of system errors and geometric aberrations such as distorted spatial perception (Cutolo et al., 2018). Though video and overlay may be well synchronised, there is inevitably some delay between actual motion and perception of the motion, both real and overlaid, which can slow down surgical motion and may increase errors. Guo et al. (2019) also reported that the absence of a direct view to the real world makes surgeons nervous.

In OST-HMDs a transparent monitor displaying graphical content is located between the surgeon’s line of vision and the target organ structures. This provides an unhindered view of reality, natural stereo vision capabilities with no lag or loss of resolution associated with the real scene. The downsides, however, are dynamic registration errors for the augmented view, latency when moving, static registration errors, complex calibration and unnatural perceptual issues, such as the fact that nearer virtual objects don’t occlude real objects in the background (Rolland et al., 1995).

### 2.1 Commercial OST-HMD devices

Prior to 2013 research in OST-HMD relied largely on custom built devices. It is a technically difficult challenge to make such a device, incorporating miniaturised displays into a wearable headset with half-silvered mirrors enabling free view of the real scene. The optical setup to display a bright image with good contrast and resolution covering a wide field-of-view is technically hard to achieve. Only two of the papers in our review use custom devices.

The potential commercial benefits of being able to place graphical information directly overlaid on the wearer’s view of the real world is a vision that has led to the development of a number of commercial devices. Google Glass, released in 2013, is a lightweight monocular AR device enabling display of information while continuing daily life. The Microsoft HoloLens, released in 2017, is a larger HMD that incorporates stereo vision, low latency room mapping and head tracking as well as gesture-based interaction using only the wearer’s hands. Numerous other devices have appeared offering different levels of comfort and function (for a more detailed list of OST-HMD devices see Section 4.3).

Though none of these devices were specifically designed for surgical tasks, the potential for convenient display of information to the surgeon has led to the explosion of research detailed in this review. In common with any medical intervention, the fundamental questions concern safety and efficacy.

### 2.2 Safety

Convenient overlay of information comes with inherent risks. Where the aim is that the overlay directly guides surgery, accuracy is key. Some authors are critical of OST-HMD device accuracy. Condino et al. (2020) concluded from their quantitative study that the HoloLens should not be used for high-precision manual tasks. Carbone et al. (2020) also conclude that OST-HMDs are unsuitable for surgical guidance, suggesting that research should focus on addressing perceptual issues that play a critical role in limiting user accuracy. Fida et al. (2018) performed a systematic review of augmented reality in open surgery and conclude that such perceptual issues limit their usage to the augmentation of simple virtual elements such as models, icons or text.

Even accurately overlaid could distract from or hamper the surgeon’s view of the patient, potentially slowing the response to critical situations such as bleeding. Dilley et al. (2019) propose nearby presentation of correctly oriented but not registered models. Gesture interactions with the AR view may prove difficult to combine with the manual surgical task itself (Solovjova et al., 2019). Cognitive overload can occur if too much extra information is presented to the surgeon at the same time (Katić et al., 2015).

Cometti et al. (2018) analyzed the effects of mixed reality HMDs on cognitive and physiological functions during intellectual and manual tasks that last for 90 min. Their experiment consisted of 12 volunteers performing and manual tasks with and without the HoloLens while their physical and mental conditions (cognitive, cardiovascular and neuromuscular) were measured. They conclude that using the HoloLens is safe since it does not impact safety-critical human functionalities like balance and cognitive and physical fatigue. However, despite the positive outcome of the study, the authors also state that one of the prerequisites of a safe and effective usage of HMDs is that users should be receptive to the device.

While some of the technological limitations of OST-HMDs are currently being addressed, such as limited field of view and automatic eye-to-eye calibration (e.g. HoloLens 2, Microsoft Corporation, Redmond, USA), human-factor limitations remain the major hurdles that prevent the commercial success of OST-HMD AR solutions within surgical applications (Cutolo et al., 2018).

### 2.3 Efficacy

The fundamental advantage of augmented reality surgery lies in the convenient display of graphical, image, icon or text information directly on the surgeon’s view of the patient. There is no need to look away from the surgical scene or stop the operation to obtain potentially useful visual input.

When displaying guidance information, accuracy becomes a measure of system performance and the majority of the papers included in this review perform some accuracy or precision experiments. It is important to distinguish registration or tracking accuracy, which is often based on an external tracking or guidance system, from perceptual accuracy achieved by the AR system.

The ultimate test of efficacy would be improved patient outcome, but the systems reviewed are not currently at the stage of large scale clinical trials that would be needed to demonstrate patient benefit.

This review aims to give an overview of the current state of the art in OST-HMD assisted surgery by analysis of several components of the selected literature, including OST-HMD device, surgical speciality, surgical application context, surgical procedure, AR visualisations, conducted experiments and accuracy results. A special focus is given to the identification of human factors in each article.

## 3 Methods

### 3.1 Literature search

A systematic review was performed according to the preferred reporting items for systematic review and meta-analysis (PRISMA) guidelines Liberati et al. (2009). The literature search was conducted via a Google Scholar with the search terms [surgery “Head Mounted Display” “Augmented Reality” OR “Mixed Reality” surgery “Head Mounted Display” “Augmented Reality” OR “Mixed Reality” “optical see through” OR “Hololens” OR “Magic Leap” OR “Google Glass”]. An initial Google Scholar including all articles between 2013 and 2020 was conducted on February 21, 2020. An updated Google Scholar search for 2020 only was subsequently performed on January 27, 2021.

### 3.2 Other review papers

Since we want to analyze only original research papers, other review papers are not considered. Our search did return a number of these, however, which deserve some attention.

A general review of all areas of AR, including medical and surgical, is provided by Dey et al. (2018), who examine the usability of AR over a 10 year period. Chen et al. (2017) review medical applications of mixed reality and provide a broad taxonomy. A comprehensive review of medical AR is provided by Eckert et al. (2019) who conclude that there is no proof of clinical effectiveness as yet. Kersten-Oertel et al. (2013) give a comprehensive review using the DVV taxonomy and provide suggestions for areas that need attention, including specific overlays for important phases of the operation as well as optimisation of interaction and system validation.

We have also previously identified some of the barriers to adoption of surgical AR in general (Edwards et al., 2021). Existing comprehensive reviews of related surgical areas were found, including robotics (Qian et al., 2020) and laparoscopic surgery (Bernhardt et al., 2017). Orthopaedics is the dominant application area in this review and three other reviews cover this specific field well (Laverdière, Corban, Khoury, Ge, Schupbach, Harvey, Reindl, Martineau, 2019, Jud, Fotouhi, Andronic, Aichmair, Osgood, Navab, Farshad, 2020, Verhey, Haglin, Verhey, Hartigan, 2020).

It is worth noting that nearly all the review papers suggest the potential of AR in surgical applications, but cite technological hurdles to user acceptability and the lack of any clinical validation. None of these reviews cover OST-HMDs specifically, which is an increasingly popular choice. We aim to provide a critical analysis of the important characteristics of OST-HMDs, looking specifically at human factors issues, which emerged as significant area potentially limiting user acceptability of systems.

### 3.3 Literature analysis strategy

In order to narrow down the publication year search range and focus on more recent research, the number of publications resulting from the google scholar search terms in the last 20 years were analyzed (Fig. 1), which shows a steady increase from 2013, coinciding with the release of Google Glass. Due to the clear increase from that time, we chose 2013 as the starting year for our literature review.

**Fig. 2:**
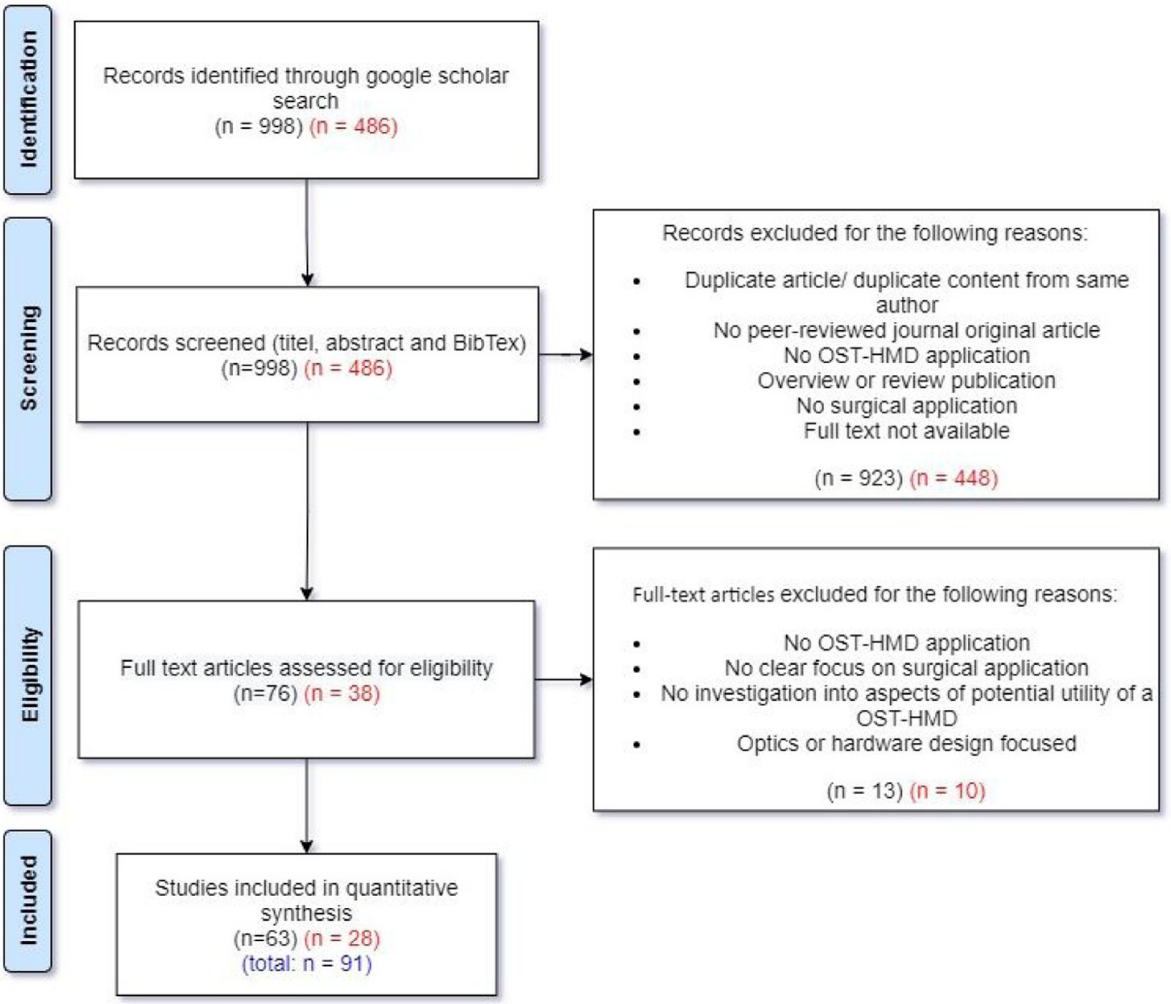
Systematic review search strategy

The review process is shown in Fig. 2 and includes the results from both the original search (February 21, 2020, numbers in black colour) and the updated search (January 21, 2021, numbers in red colour). The Google Scholar search initially resulted in 998 (486) records. In a subsequent screening phase, title, abstract and BibTex information were read to decide whether the record seems to be a relevant publication. We exclude records that are either a duplicate or contain duplicated content from the same authors compared to another record. A total of 15 (7) duplicates were excluded. During the screening the following inclusion criteria were used: The article has to 1.) be a peer-reviewed original journal article, 2.) describe an OST-HMD focused application with surgical context, 3.) is not an overview or systematic review publication (which are considered separately). Records whose full text wasn’t available were excluded. 907 (441) records that didn’t meet the inclusion criteria were excluded. Together with the 15 (7) excluded duplicates, a total of 923 (448) records were excluded during the screening phase, which led to 76 (38) remaining full text articles that were assessed for eligibility. Full text articles had to meet the following inclusion criteria: The article 1.) describes the usage of an OST-HMD, 2.) with a clear focus on a surgical application, 3.) investigates the potential utility of OST-HMDs in surgical settings and 4.) is neither optics nor hardware design focused. 13 (10) articles that didn’t meet these inclusion criteria were excluded. The remaining 63 + 28 = 91 studies that met all predefined inclusion criteria form the final set of papers examined in this review.

When reporting the results, the PRISMA guidelines were followed. Due to the inherent characteristics of the studies (small case series, subjective qualitative assessments, no controlled randomised trials) a meta-analysis could not be performed. Therefore, publication bias could not be reduced and should be taken into account.

Data extracted from the included publications were 1. Clinical setting (surgical speciality, surgical application context, surgical procedure), 2. The assessed OST-HMD device, 3. Methods (AR visualisations, Conducted experiments), 4. Key results (Accuracy), 5. Human factors

## 4 Analysis of the literature search

This section summarises the results of the included 91 articles that were identified in the literature review process. An overview of used OST-HMD device, surgical application context and surgical procedure can be found in Table 2. Appendix Table A.1 contains details about AR visualisations, conducted experiments and accuracy results.

**Table 1:** Distribution of the included articles per surgical speciality.

Surgical speciality	Articles
Orthopaedic Surgery	Armstrong et al. (2014), Ponce et al. (2014), Chen et al. (2015), Wang et al. (2016), Stewart and Billinghurst (2016), Hiranaka et al. (2017), Jalaliniya et al. (2017), Unberath et al. (2018), El-Hariri et al. (2018), Deib et al. (2018), Andress et al. (2018), Condino et al. (2018), Gibby et al. (2019), de Oliveira et al. (2019), Aaskov et al. (2019), Liebmann et al. (2019), Fotouhi et al. (2019a), Fotouhi et al. (2019b), Pietruski et al. (2020), Laguna et al. (2020), Gibby et al. (2020), Gu et al. (2020), Viehöfer et al. (2020), Dennler et al. (2020), Kriechling et al. (2020), Matsukawa and Yato (2020)
General Surgery	Lin et al. (2018), Wu et al. (2018), Mahmood et al. (2018), Rojas-Muñoz et al. (2019), Li et al. (2019), Pelanis et al. (2020), Zhou et al. (2020), Al Janabi et al. (2020), Jud et al. (2020), Galati et al. (2020), Li et al. (2020a)
Neurosurgery	Yoon et al. (2017), Karmonik et al. (2018), Frantz et al. (2018), Nguyen et al. (2020), Zhang et al. (2019), Heinrich et al. (2019), Baum et al. (2020), Liounakos et al. (2020), Sun et al. (2020b)
Vascular Surgery	Kaneko et al. (2016), Kuhlemann et al. (2017), Zhou et al. (2019b), Rynio et al. (2019), Rojas-Muñoz et al. (2020a), Park et al. (2020), Mendes et al. (2020)
General surgical applications	Meulstee et al. (2019), Chien et al. (2019), Boillat et al. (2019), Guo et al. (2019), Dallas-Orr et al. (2020), Luzon et al. (2020), Cartucho et al. (2020), Kumar et al. (2020)
Dental Surgery	Katić et al. (2015), Liebert et al. (2016), Song et al. (2018), Zhou et al. (2019a), Zafar and Zachar (2020)
Heart Surgery	Li et al. (2017), Zou et al. (2017), Brun et al. (2019), Liu et al. (2019)
Otolaryngology - head and neck surgery	Rojas-Muñoz et al. (2020b), Gnanasegaram et al. (2020), Scherl et al. (2020), Creighton et al. (2020),
Reconstructive Surgery	Mitsuno et al. (2017), Pratt et al. (2018), Jiang et al. (2020)
Maxillofacial Surgery	Pietruski et al. (2019), Pepe et al. (2019), Sun et al. (2020a)
Urological surgery	Borgmann et al. (2016), Dickey et al. (2016), Schoeb et al. (2020)
Robot-assisted surgery	Qian et al. (2018), Fotouhi et al. (2020)
Paediatric surgery	Wellens et al. (2019), Fitski et al. (2020)
Visceral Surgery	Sauer et al. (2017)
Interventional Oncology	Li et al. (2020b)
Laparoscopic Surgery	Zorzal et al. (2020)
Anaesthesiology	Schlosser et al. (2019)

**Table 2:** Studies listed by OST-HMD, Surgical context and surgical procedure : Acronyms: SG: Surgical guidance. PS: Preoperative surgical planning. SAA: Intraoperative surgical anatomy assessment. ST: Surgical training. REV: Intraoperative review of preoperative 2D imaging and/or patient records. TELC: Teleconsultation during surgery. TELM: Telementoring. DOC: Intraoperative documentation. PM: Patient monitoring. Acronyms for surgical guidance applications: TP: Surgical tool placement. IO: Image overlay for navigation. SI: Screw insertion. NI: Needle insertion. CI: Catheter insertion. KWI: K-Wire insertion. EG: MIS Endoscopy guidance. SP: Stent-graft placement. DTG: Drill trajectory guidance. PN: Imaging probe navigation. SNN: Surgical saw navigation. CA: C-arm positioning guidance. RP: robot placement. DG: dissection guidance. AI: anatomy identification.

Study	OST-HMD	Surgical context	Surgical procedure
Armstrong et al. (2014)	Google Glass	TELC	Reconstructive limb salvage procedures
Ponce et al. (2014)	Google Glass	TELM	shoulder arthroplasty
Chen et al. (2015)	nVisor ST60	SG (SI)	Percutaneous implantation of sacroiliac joint screw
Katić et al. (2015)	Custom Device	SG (DTG)	Dental implant surgery
Borgmann et al. (2016)	Google Glass	REV, ST, DOC, TELC	Different urological surgical procedures
Dickey et al. (2016)	Google Glass	ST, TELM	Inflatable penile prosthesis placement
Liebert et al. (2016)	Google Glass	PM	Bronchoscopy
Wang et al. (2016)	nVisor ST60	SG (SI)	Percutaneous implantation of sacroiliac joint screw
Stewart and Billinghurst (2016)	Brother AirScouter WD-100G	SG (TP)	General intra-operative guidance (no concrete application, only measurement of attentiveness to the surgical field)
Kaneko et al. (2016)	Moverio BT-200	SG (CI)	Central venous catheterisation under US guidance
Yoon et al. (2017)	Google Glass	SG (SI)	spine instrumentation (pedicle screw placement)
Jalaliniya et al. (2017)	Google Glass	REV, TELC	Orthopaedic procedures
Li et al. (2017)	HoloLens	PS, ST	Preoperative diagnosis & planning of coronary heart disease
Kuhlemann et al. (2017)	HoloLens	SG (CI)	Interventional endovascular stenting of aortic aneurysm
Sauer et al. (2017)	HoloLens	SAA, TELC	Visceral-surgical interventions
Mitsuno et al. (2017)	Moverio BT-200	SAA	Improvement of the body surface contour in plastic surgery.
Hiranaka et al. (2017)	PicoLinker glasses	SG (KWI)	Fluoroscopy controlled K-wire insertion into femur
Zou et al. (2017)	Custom Device	PS	Preoperative diagnosis of coronary heart disease
Deib et al. (2018)	HoloLens	SG (NI)	Percutaneous vertebroplasty, kyphoplasty and discectomy procedures
Andress et al. (2018)	HoloLens	SG (KWI)	Percutaneous orthopaedic surgical procedures
Song et al. (2018)	HoloLens	SG (DTG)	Access cavity Preparation in Endodontic treatment
Condino et al. (2018)	HoloLens	ST	Hip arthroplasty
Qian et al. (2018)	HoloLens	SG (RP, TP, EG)	Increase the First Assistant’s task performance during robot-assisted laparoscopic surgeries
El-Hariri et al. (2018)	HoloLens	SG (TP)	Intra-operative bone localisation
Karmonik et al. (2018)	HoloLens	PS	Identification of a hemodynamic scenario that predicts an aneurysm rupture
Lin et al. (2018)	HoloLens	SG (NI)	Needle biopsy
Frantz et al. (2018)	HoloLens	SG (IO)	Neurosurgical applications
Pratt et al. (2018)	HoloLens	SG (DG)	Vascular pedunculated flaps of the lower extremities (reconstruction surgery)
Unberath et al. (2018)	HoloLens	SG (CA)	percutaneous orthopaedic procedures
Mahmood et al. (2018)	HoloLens	ST	example: transesophageal echocardiography examination
Wu et al. (2018)	HoloLens	SG (IO)	N/A
Boillat et al. (2019)	Google Glass	DOC	Surgical time-out checklist execution
Meulstee et al. (2019)	HoloLens	SG (TP)	N/A
Gibby et al. (2019)	HoloLens	SG (SI)	pedicle screw placement
Brun et al. (2019)	HoloLens	PS	Repair for complex congenital heart disease
de Oliveira et al. (2019)	HoloLens	SG (IO)	Orthopaedic surgery (no specific procedure)
Fotouhi et al. (2019a)	HoloLens	SG (KWI)	C-arm fluoroscopy guided k-wire placement
Aaskov et al. (2019)	HoloLens	SG (AI)	Identification of spinal anatomy underneath the skin
Guo et al. (2019)	HoloLens	SG (IO)	General image-guided surgical navigation (no specific application)
Liebmann et al. (2019)	HoloLens	SG (SI)	Placement of pedicle screws in spinal fusion surgery
Liu et al. (2019)	HoloLens	SG (CI)	transcatheter procedures for structural heart disease
Rojas-Muñoz et al. (2019)	HoloLens	TELM	Abdominal incision
Rojas-Muñoz et al. (2020a)	HoloLens	TELM	Leg fasciotomy
Li et al. (2019)	HoloLens	SG (TP)	liver tumor puncture
Pepe et al. (2019)	HoloLens	SG (IO)	Head and neck tumor resections
Zhou et al. (2019b)	HoloLens	SG (NI)	Seed implantation thoracoabdominal tumor brachytherapy
Chien et al. (2019)	HoloLens	SG (IO)	General SG (no specific surgical application)
Zhang et al. (2019)	HoloLens	SG (TP)	Craniotomy
Heinrich et al. (2019)	HoloLens	SG (NI)	Needle-based spinal interventions
Wellens et al. (2019)	HoloLens	PS	Nephron-sparing surgery
Fotouhi et al. (2019b)	HoloLens	SG (TP)	Percutaneous orthopaedic treatments
Rynio et al. (2019)	Hololens	SG (SP)	Endovascular aortic repair
Zhou et al. (2019a)	Magic Leap One	SG (PN)	Tooth decay management
Pietruski et al. (2019)	Moverio BT-200	SG (SSN)	Mandibular resection
Schlosser et al. (2019)	Vuzix M300	PM	None
Fotouhi et al. (2020)	HoloLens	RP	Set up of robotic arms by surgical staff (especially minimally invasive gastrectomy (abdominal surgery))
Pelanis et al. (2020)	HoloLens	PS	Liver resection
Nguyen et al. (2020)	HoloLens	SG (IO)	Neurosurgical applications
Zhou et al. (2020)	HoloLens	SG (NI)	Seed implantation thoracoabdminal brachytherapy
Baum et al. (2020)	HoloLens	ST	Neurosurgical burr hole localisation
Al Janabi et al. (2020)	HoloLens	SG (EG)	Ureteroscopy
Pietruski et al. (2020)	Moverio BT-200	SG (SSN)	Free fibula flap
Liounakos et al. (2020)	Moverio BT-300	SG (EG)	Percutaneous endoscopic lumbar discectomy
Gnanasegaram et al. (2020)	HoloLens	ST	N/A
Sun et al. (2020b)	HoloLens	SG(CI)	External ventricular drainage (EVD)
Park et al. (2020)	HoloLens	PS	Endovascular procedures
Mendes et al. (2020)	Arzyon headset	ST	Central venous catheterisation
Laguna et al. (2020)	HoloLens	PS	Repair of complex paediatric elbow fractures
Dallas-Orr et al. (2020)	HoloLens	PS	Complex surgical procedures
Zafar and Zachar (2020)	HoloLens	ST	No direct surgical procedure (teaching of dental anatomy)
Fitski et al. (2020)	HoloLens	PS	Nephron-Sparing Surgery in Wilms’ Tumor Surgery
Schoeb et al. (2020)	HoloLens	ST	Urologic surgical procedures (bladder catheter placement)
Luzon et al. (2020)	HoloLens	SG (DG)	Right colectomy with extended lymphadenectomy
Matsukawa and Yato (2020)	PicoLinker glasses	SG (SI)	Single-segment posterior lumbar interbody fusion
Yang et al. (2020)	HoloLens	SG (NI)	Transjugular intrahepatic portosystemic shunt (TIPS)
Li et al. (2020b)	HoloLens	SG (NI)	Percutaneous needle interventions
Kumar et al. (2020)	HoloLens	PS	Example use cases: laparoscopic liver resection and congenital heart surgery
Li et al. (2020a)	HoloLens	SG (DG), PS, TELC, ST	Laparoscopic partial nephrectomy / Laparoscopic radical nephrectomy
Gibby et al. (2020)	HoloLens	SG (NI)	Percutaneous image-guided spine procedures
Gu et al. (2020)	HoloLens	SG (DTG)	Total shoulder arthroplasty
Galati et al. (2020)	HoloLens	SAA	Open Abdomen Surgery
Viehöfer et al. (2020)	HoloLens	SG (SNN)	Hallux Valgus correction
Dennler et al. (2020)	HoloLens	SG (SI)	Spinal instrumentation
Kriechling et al. (2020)	HoloLens	SG (KWI)	Reverse total shoulder arthroplasty (RSA)
Zorzal et al. (2020)	Metavision Meta 2	SG (EG)	Laparoscopic procedures
Cartucho et al. (2020)	HoloLens	SAA	N/A
Rojas-Muñoz et al. (2020b)	HoloLens	TELM	Cricothyroidotomy
Scherl et al. (2020)	HoloLens	SG (IO)	Surgery of the parotid gland
Creighton et al. (2020)	HoloLens	SG (IO)	Lateral Skull Base Surgery
Jiang et al. (2020)	HoloLens	SG (DG)	Perforator flap transfer
Sun et al. (2020a)	HoloLens	SG (IO)	Mandibular reconstruction

**Table 4:** Identified Human Factors, grouped into the categories 1.) Information Perception, 2.) Cognitive Processing and 3.) Control Actions.

Abbreviation	Human Factor
**Information Perception**	
SPATIAL_PERC	Spatial perception/awareness
INC	Inconvenience
DPPC	Missing/impaired depth perception
EYE	Individually different visual processing capabilities between dominant and non-dominant eye
COMF	Perceived comfort level when wearing OST-HMD
PER_REAL_AUG	Perception of spatial relationships between real and virtual objects
IMMR	Personal degree of perceived immersion
**Cognitive Processing**	
ATTN_SHIFT	Attention switch between surgical site and separate computer monitor
MM	Error-prone and cognitively demanding mental mapping of 2D image data to 3D word
SLC	Steep learning curve
EXP_OUTCOME	Influence of clinician’s experience on surgical outcome
DIST	Distraction
INTPN_2D_DETAIL	Risk of incorrect interpretation of 2D image details
INTRA_OP_NAV	Impaired intraoperative navigation abilities due to absence of visual aids
COMM_3D	Personal 3D anatomical imagination capabilities affect communication between experts
CONF	Confidence
FRUS	Frustration
SUBJ_MEAS_OUTCOME	Subjective measurement of surgical outcome
EASE_HCI	Perceived degree of ease and intuitiveness of HCI
CLIN_EXP_2D	Dependence on clinical experience for interpretation of 2D image data
EMP_EST_2D	Inaccurate empirical estimation of target locations in 2D anatomy images
ANAT_PLN	Impaired anatomical understanding during preoperative planning due to 2D imaging data
CONC_LS	Loss of concentration
MIP	Limited mental information processing abilities
STRESS	Experience of stress
ENG_MOT	Engagement and motivation
PREF_HOL	Preferred degree of superimposition of 3D objects onto the surgical field (precise vs shifted superimposition)
USEF	Perceived usefulness of OST-HMD
ANX	Anxiety
**Control Actions**	
VIS_OPT	Selection of preferred mode of visualisation
SURG	Increased risk of surgical error
HEC	Unfamiliar/cognitively demanding hand-eye coordination
TOOL_ADJUST	Error-prone manual tool adjustment
FAT	Visual Fatigue

### 4.1 Annual distribution of selected articles

**Fig. 3:**
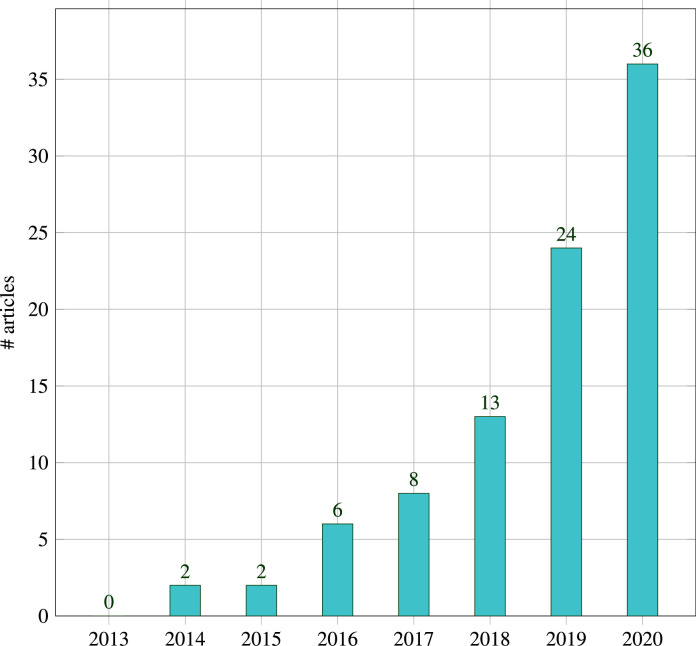
Systematic review results overview: Annual Distribution of selected 91 studies from 2013-2020

Fig. 3 shows the annual distribution of the 91 studies during 2013-2020. There were no articles in the year 2013 that fulfilled the inclusion criteria. Starting from 2014 there has been a steady increase in the number of publications. The increasing trend tends to be related to the release of major OST-HMDs like Google Glass and Microsoft HoloLens, and will be discussed in more detail in Section 4.3.

### 4.2 Surgical speciality

**Fig. 4:**
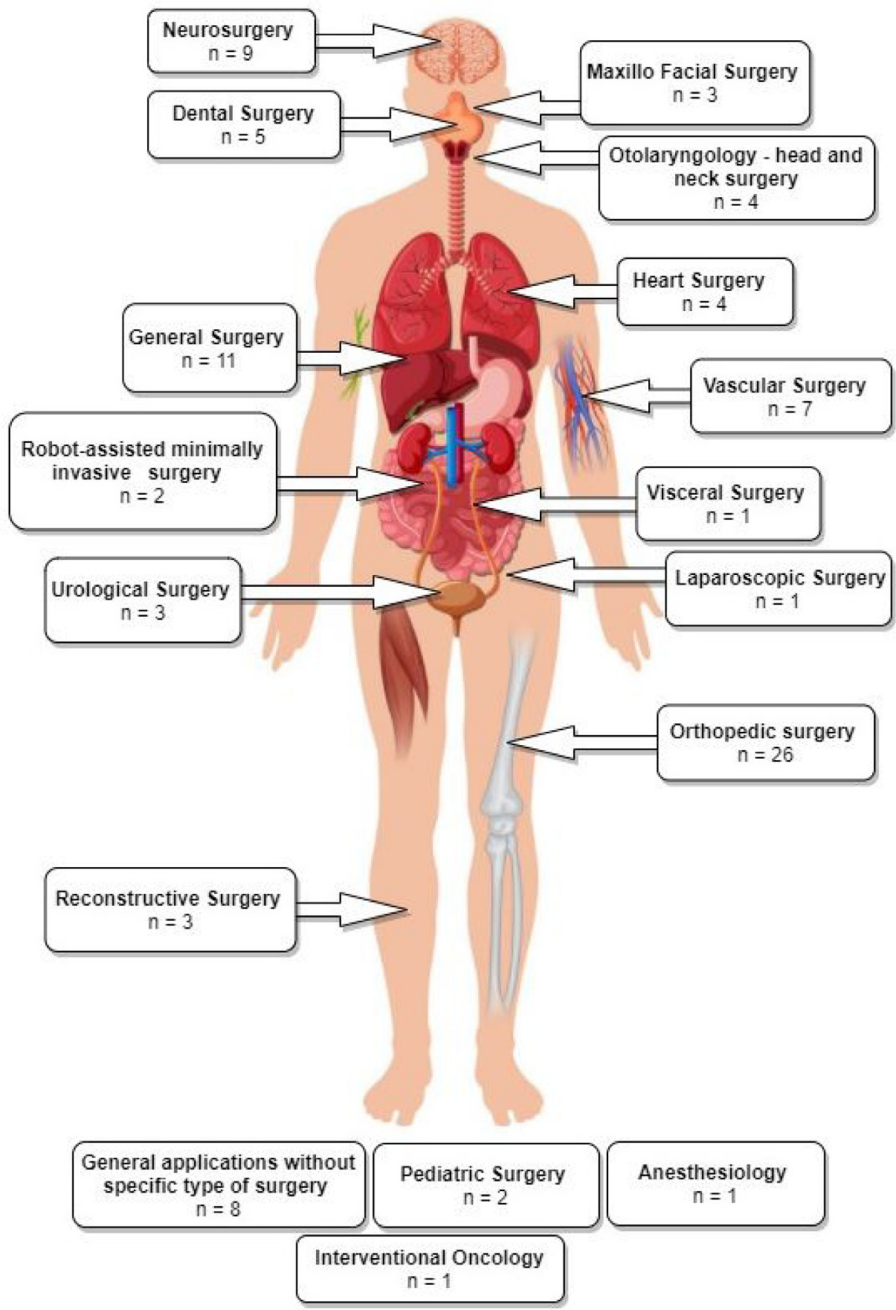
Graphical illustration of included articles grouped by surgical speciality and placed at respective human body regions

**Fig. 5:**
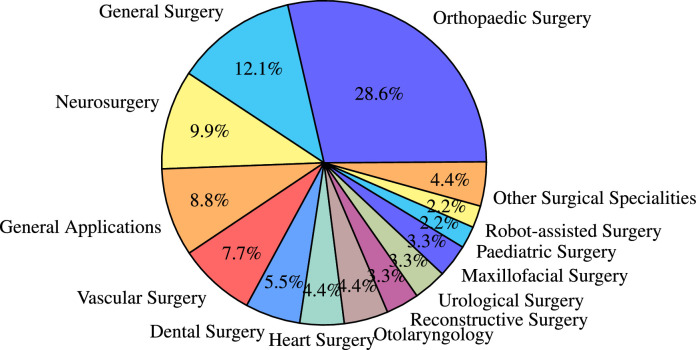
Pie chart showing the distribution of the included 91 papers among the identified surgical specialities

We found that OST-HMDs have been applied in a variety of surgical specialities. Fig. 4 shows a graphical illustration of all articles grouped into their surgical speciality and placed at their respective body region. Fig. 5 shows the proportion of publications for each surgical speciality. Orthopaedic surgery dominates (28.6%, n=26), perhaps since proximity to bone requires only rigid registration and somewhat lower accuracy is required compared to applications such as neurosurgery. General surgery, neurosurgery, applications without a concrete surgical speciality and vascular surgery follow with more than five articles each. Dental surgery is represented with five articles, followed by heart surgery and Otolaryngology (n=4 each). Other surgical specialities include reconstructive surgery, urology and maxillofacial surgery (n=3 each). A few attempts have been made to explore potential benefits of OST-HMDs in robot-assisted surgery and paediatric surgery (n=2 each). Interventional oncology, laparoscopic surgery, visceral surgery and anaesthesiology are represented with one article. Specific articles per surgical speciality are detailed in Table 1. While orthopaedics still dominates, other applications, including general, vascular and neurosurgery, are increasingly represented in the latter half of the survey period as interest in AR applications spreads to other surgical fields.

### 4.3 Optical see-through head-mounted displays (OST-HMDs)

**Fig. 6:**
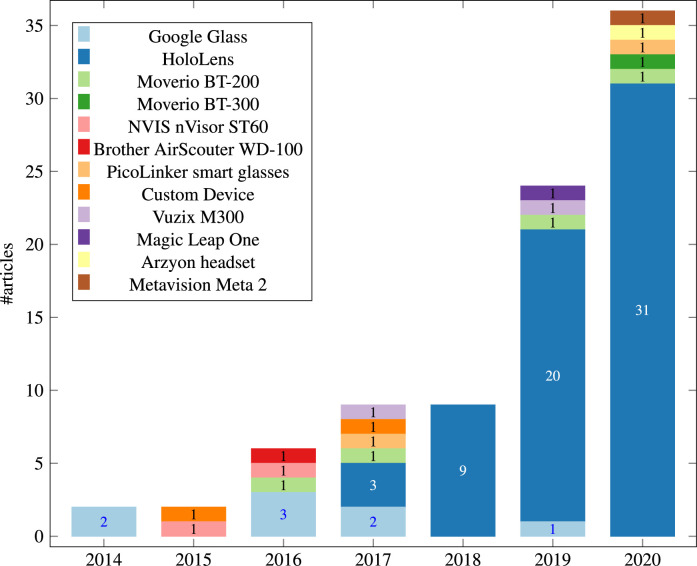
Annual Distribution of articles by **OST-HMD** device from 2014-2020

Fig. 6 depicts the annual distribution by OST-HMD between 2014–2000. Google Glass, the device with the second highest number of articles (n=8), dominates the distribution in 2014, but interest decreases from 2015 to 2017, perhaps due to diminishing support from Google. The Microsoft HoloLens was released in 2016 and has dominated the field of OST-HMD assisted surgery since then, with a steady increase in papers from 2017 and accounting for the majority of articles (n=66). Following HoloLens and Google Glass, the Moverio BT-200, which was released in 2014, has the third highest number of articles (n=4) and was used once in 2016, 2017, 2019 and 2020. Its successor, the Moverio BT-300, was released in late 2016 and has only one application in 2020. The Magic Leap One, released in 2018 and attracting huge initial investment, has not established itself in OST-HMD assisted surgery, generating only one article in 2019. Other devices include the NVIS nVisor ST (n=2), Vuzix M300, Brother AirScouter WD-100, Aryzon headset, Metavision Meta 2 and PicoLinker (n=1 each).

To summarise, the HoloLens clearly dominates the field, but there is interest in other devices such as Moverio BT. This is a rapidly developing field at present and we can expect further devices to appear on the market in the next few years.

## 5 Surgical application context

**Fig. 7:**
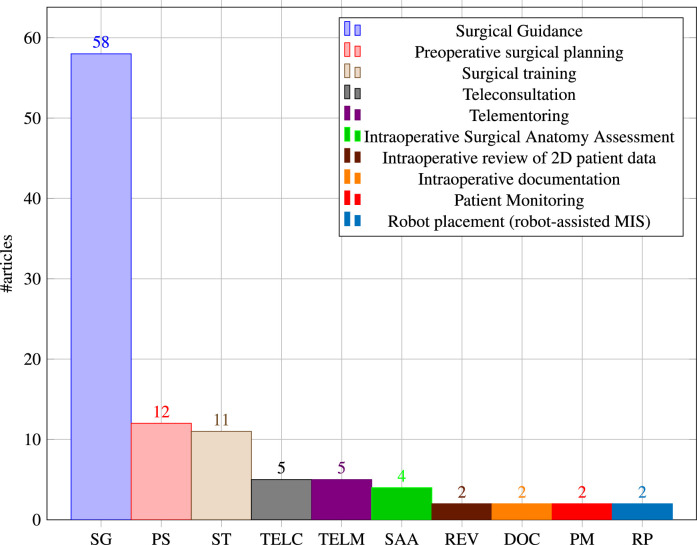
Distribution of articles by **surgical application context**

Surgical application contexts define how OST-HMD assistance is intended to improve surgical practice. Fig. 7 shows the distribution of all identified contexts. Surgical guidance is by far the most popular (n=58), followed by preoperative surgical planning (n=12) and surgical training (n=11) then Teleconsultation and telementoring (n=5 each). Four articles were included where the surgeon views a 3D patient anatomy hologram to aid clinical decision making rather than intraoperative guidance, which we called *intraoperative surgical anatomy assessment*. The remaining applications that have been identified are intraoperative review of preoperative 2D imaging and/or patient records, intraoperative documentation, patient monitoring and preparation of robot-assisted MIS (n=2 each). We expand on some of the surgical application contexts and respective articles in the following subsections.

### 5.1 Surgical guidance

We use the definition of surgical guidance or image-guided surgery from Cleary and Peters (2010): a medical procedure in which a surgeon uses computer-based virtual pre- or intraoperative image overlays to visualise and target patient anatomy. They also state that an image-guided intervention includes registration and tracking methods, but we also consider an OST-HMD based solution to be of the category image guidance if it uses registered holographic image overlays without tracking if these overlays support a clinician in visualizing and targeting the surgical site.

**Fig. 8:**
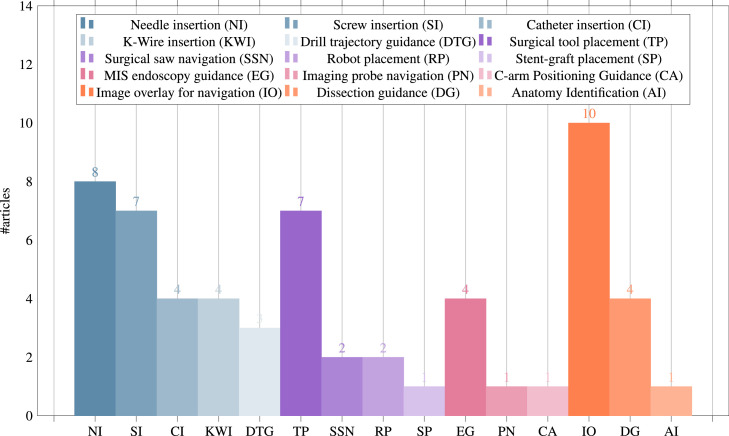
Surgical guidance applications: Distribution of the subset of final 91 articles (n=59) by applications of **surgical guidance**, grouped into the four categories 1. navigation of a linear path, 2. navigation of surgical tools or equipment, 3. navigation of an imaging device, 4. general guidance to help spatial awareness not associated with a specific task

Since this broad definition covers over half the included papers, we further split OST-HMD assisted surgical guidance into different applications, whose distribution is presented in Fig. 8. General image overlay for navigation systems (n=10) overlay a registered 3D anatomy model in order to provide surgical guidance, including applications in neuronavigation (Frantz, Jansen, Duerinck, Vandemeulebroucke, 2018, Nguyen, Cardinell, Ramjist, Lai, Dobashi, Guha, Androutsos, Yang, 2020), orthopaedic procedures (de Oliveira et al., 2019), algorithm-focused registration approaches (Wu, Chien, Wu, Lee, 2018, Aaskov, Kawchuk, Hamaluik, Boulanger, Hartvigsen, 2019, Chien, Tsai, Wu, Lee, 2019) and maxillo-facial tumor resection (Pepe et al., 2019).

**Fig. 9:**
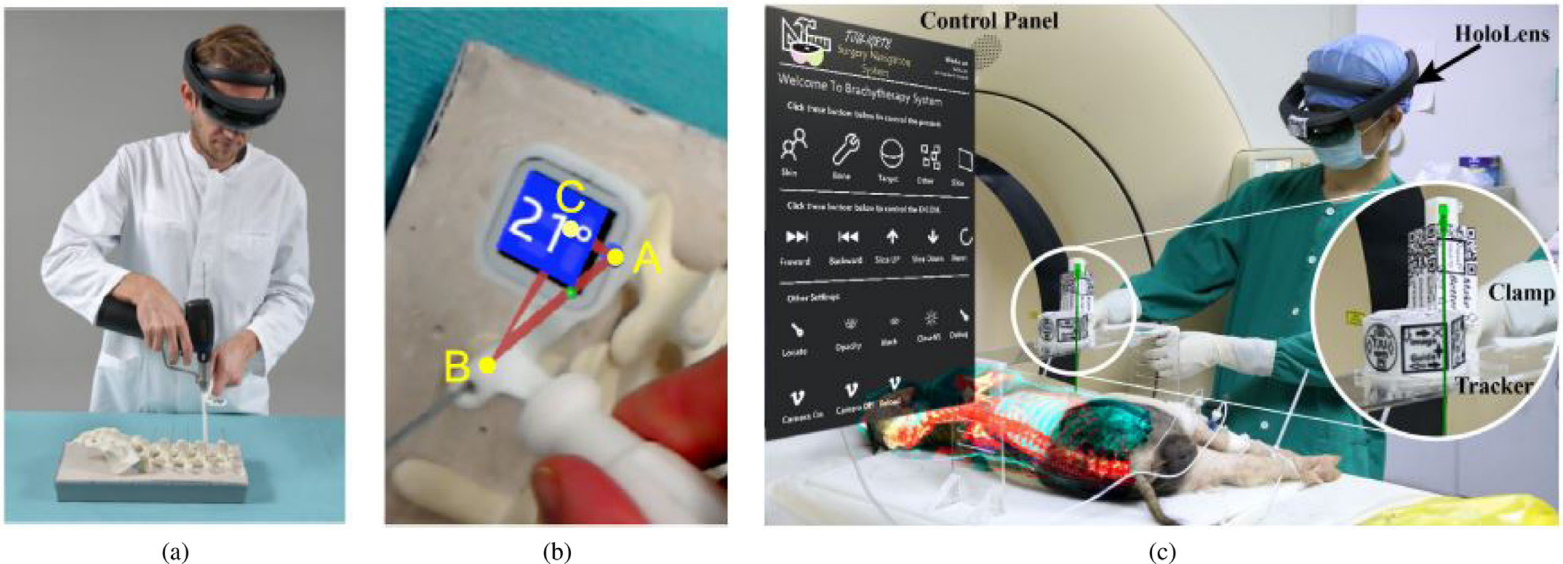
Guided screw insertion and needle insertion examples. (a) A surgeon uses a custom-made navigation device in an experimental setup (b). Augmented drill entry points (shown in blue) are used to start the navigation. During the guided drill procedure, the 3D angle between current and targeted screw trajectory and their deviation angle are displayed. *(source:**Liebmann et al. (2019)*Fig. 5*b and 5d)*. (c) Mixed reality needle insertion navigation system for low dose-rate (LDR) brachytherapy *(source:**Zhou et al. (2019b)**) Fig. 1*.

Needle insertion (n=8) has emerged as an application since 2018, mostly using the HoloLens, and was investigated in percutaneous spine procedures (Deib et al., 2018), needle biopsy (Lin et al., 2018), thoracoabdominal brachytherapy (Zhou, Yang, Jiang, Zhang, Yan, 2019, Zhou, Yang, Jiang, Zhang, Yan, Ma, 2020) and needle-based spinal interventions (Heinrich et al., 2019). Zhou et al. (2019b) presented a mixed reality based needle insertion navigation system for low-dose-rate brachytherapy that was tested in animal (Fig. 9 (c)) and phantom experiments. Reported benefits of this needle insertion approach include clinically acceptable needle insertion accuracy and a reduction of the number of required CT scans.

Tool placement examples (n=7) include investigated attentiveness to the surgical field during navigation (Stewart and Billinghurst, 2016), a first assistant’s task performance during robot-assisted laparoscopic surgery based tool manipulation (Qian et al., 2018), bone localisation (El-Hariri et al., 2018), an optical navigation concept (Meulstee et al., 2019), liver tumor puncture (Li et al., 2019), craniotomy assistance (Zhang et al., 2019) and percutaneous orthopaedic treatments (Fotouhi et al., 2019b).

OST-HMD assisted screw insertion (n=7) has been explored with different holographic visualisations. Yoon et al. (2017) presented an application for pedicle screw placement in spine instrumentation that streamed 2D neuronavigation images onto a Google Glass. Surgeons reported an overall positive AR-experience. Liebmann et al. (2019) developed a HoloLens pedicle screw placement approach for spinal fusion surgery that uses holopgraphic 3D angles between current and targeted screw trajectory, using deviation in angle to guide the surgeon (Fig. 9 (a) and (b)). The reported results of a lumbar spine phantom experiment indicate a promising screw insertion accuracy with the caveat that surrounding tissue was not taken into account. Other articles describing pedicle screw insertion include Yoon et al. (2017) and Gibby et al. (2019). Percutaneous implantation of sacroiliac joint screws is presented in Chen et al. (2015) and Wang et al. (2016).

**Fig. 10:**
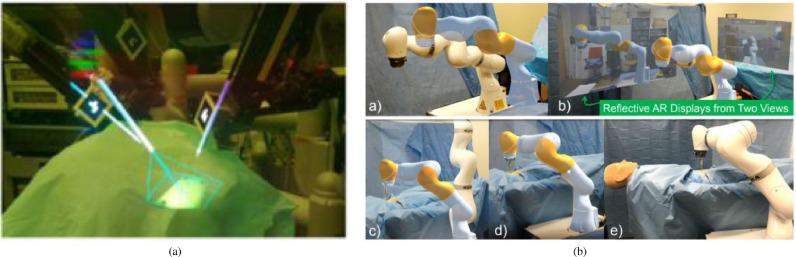
(a) Robotic instrument placement and endoscopy guidance: Navigation aids for the first assistant: Real-time renderings of a robotic endoscope and robotic instruments that are superimposed on their physical counterparts. In addition, endoscopy guidance is realised via an endoscopy visualisation being registered with a viewing frustrum *(source:*Fig. 4*(f) of**Qian et al. (2018)**)* (b) Robot placement: Reflective-AR Display aided alignment between a real robot arm and its virtual counterpart and subsequent robot placement to its intended position in preparation for robotic surgery *(source.*Fig. 4*of**Fotouhi et al. (2020)**)*

Catheter insertion (n=4) also has to deal with the manipulation of flexible structures and has been applied to US-guided central venous catheterisation (Kaneko et al., 2016), radiaton-free endovascular stenting of aortic aneurysm (Kuhlemann et al., 2017) and transcatheter procedures for structural heart disease (Liu et al., 2019). K-wire insertion in orthopaedic procedures (n=4) was addressed by experiments investigating fluoroscopy controlled wire insertion into femur (Hiranaka et al., 2017), percutaneous orthopaedic surgical procedures (Andress et al., 2018) and C-arm fluoroscopy guidance (Fotouhi et al., 2019a). The exploration of potential benefits of holographic camera views for endoscopy guidance (n=4) has been conducted in first assistant support in robot-assisted laparoscopic surgery (Qian et al., 2018) (Fig. 10 (a)), percutaneous endoscopic lumbar discectomy (Liounakos et al., 2020) and ureteroscopy (Al Janabi et al., 2020).

Drill trajectory guidance (n=3) explores potential advantages of holographic guidance information such as drill angle and deviation between actual and planned drill path and has been used in dental implant surgery (Katić et al., 2015) and endodontic treatments (Song et al., 2018). Surgical saw navigation using holographic cutting guides (n=2) was presented in mandibular resection (Pietruski et al., 2019) and free fibula flap harvest (Pietruski et al., 2020).

**Fig. 11:**
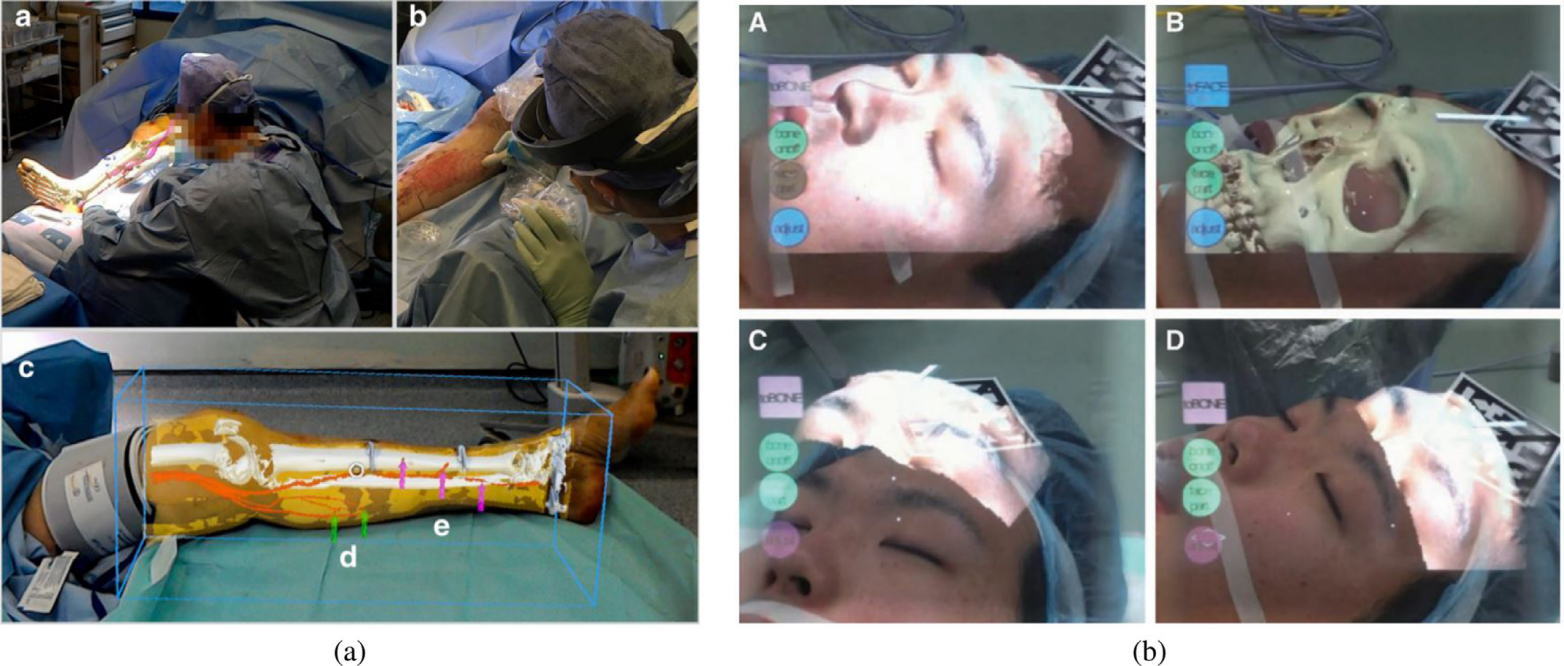
(a) Dissection Guidance example in reconstructive surgery: HoloLens based identification of vascular pedunculated flaps: a CTA-based 3D model of a female patient’s leg consisting of segmented skin, bone, bone, vessels and vascular perforators lower leg is superimposed on the patient anatomy. The surgeon confirms perforator location with audible Doppler ultrasonography *(source:*Fig. 3*of**Pratt et al. (2018)**)* (b) Surgical Anatomy Assessment example in plastic surgery: AR views of the Moverio BT-200 smart glasses showing a patient with osteoma and holographic facial anatomy (face surface and facial bones including the osteoma) superimposed onto a patient’s face *(source:*Fig. 8*of**Mitsuno et al. (2017)**)*

In addition to surgeons themselves, other clinical staff in the operating theatre can benefit from OST-HMD assitance. In minimally invasive robotic surgery it is usually the first assistant’s responsibility to set up the robot arms prior to intraoperative robot control conducted by a surgeon. We identified 2 articles that present HoloLens applications aiming to support the first assistant during robot-assisted surgery: 1.) Qian et al. (2018) robotic instrument placement in laparoscopic surgery from (Fig. 10(a)) and 2.) full robot arm placement in minimally invasive gastrectomy (abdominal surgery) from Fotouhi et al. (2020) (Fig. 10(b)). The remaining applications of surgical guidance cover topics such as stent-graft placement in endovascular aortic repair (Rynio et al., 2019), imaging probe navigation for tooth decay management (Zhou et al., 2019a), C-arm positioning guidance in percutaneous orthopaedic procedures (Unberath et al., 2018), identification of spinal anatomy underneath the skin (Aaskov et al., 2019) and dissection guidance for vascular pedunculated flaps of the lower extremities presented by Pratt et al. (2018) (Fig. 11(a)). A HoloLens based mixed reality approach was decised in which the surgeon has to manually register a CTA-based 3D model of a patient’s leg to the respective patient anatomy using HoloLens hand gesture and voice command interaction. After a surgical patient case study, surgeons confirmed that this mixed reality solution is more reliable and less time consuming than audible Doppler ultrasound which is the conventional non-AR method.

With the main research focus being image-guidance, it is very important to consider safety and accuracy in such systems. Overconfidence in the accuracy of guidance or visual clutter of the viewed scene may lead to an increase in surgical errors and a careful balance needs to be struck to provide useful information rather than cognitive overload.

### 5.2 Other surgical application contexts

Preoperative planning applications from Zou et al. (2017) and Li et al. (2017) addressed human-computer interaction issues of conventional approaches in preoperative diagnosis of coronary heart disease that lead to inaccurate diagnosis results and propose a hand gesture based interactive holographic diagnosis system aiming to provide a natural and intuitive interaction. Karmonik et al. (2018) used holographic 3D vascular structures to improve the extraction and communication of complex MRI image data in the context of aneurysm rupture prediction. Pelanis et al. (2020) addressed planning of liver resection surgery and found that 3D holographic liver anatomy visualisations improve the user’s spatial understanding. Other articles that were categorised as preoperative surgical planning investigate potential planning improvements for repair of complex congenital heart disease (Brun et al., 2019) and preoperative anatomy assessment for nephron-sparing surgery (Wellens et al., 2019).

**Fig. 12:**
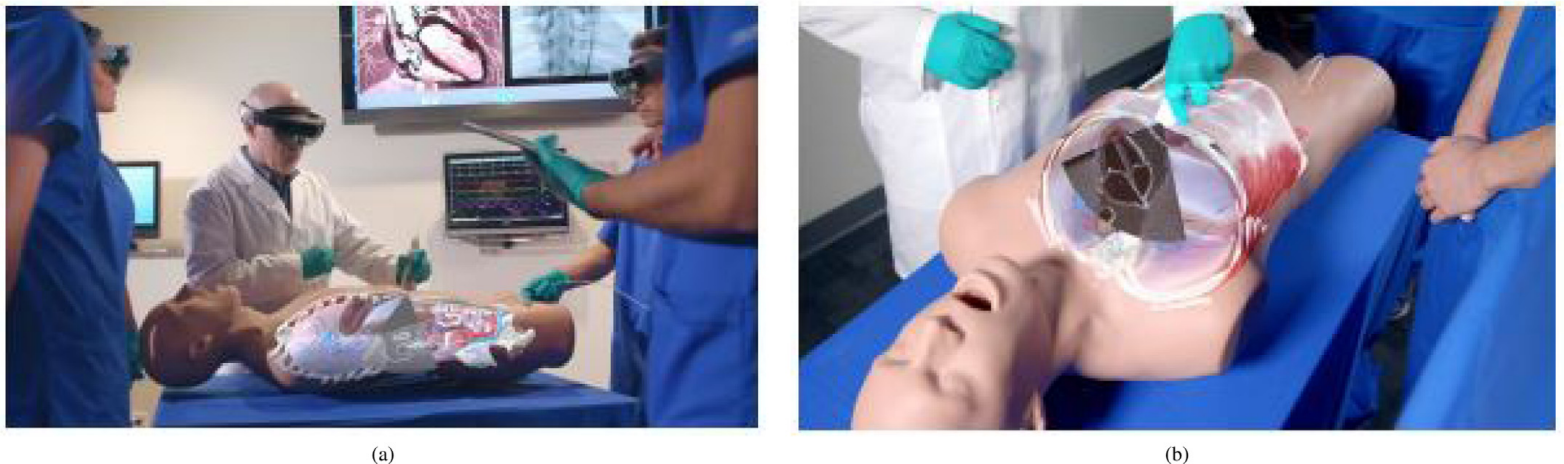
Surgical Training Example Application: Ultrasound Education. (a) Multiple users can see holographic anatomical cross sections mapped on a patient simulator and the ultrasound scan plane. (b) Holograhic subcostal four-chamber view coming out of the simulator probe. *Source:*Fig. 3*and 7 of**Mahmood et al. (2018)*

Benefits of OST-HMD AR during surgical training have been explored for preoperative diagnosis and planning of coronary heart disease (Li et al., 2017), intraoperative surgical tool guidance during hip arthroplasty simulation (Condino et al., 2018), neurosurgical burr hole localisation (Baum et al., 2020) and transesophageal echocardiography examination from Mahmood et al. (2018) shown in Fig. 12.

**Fig. 13:**
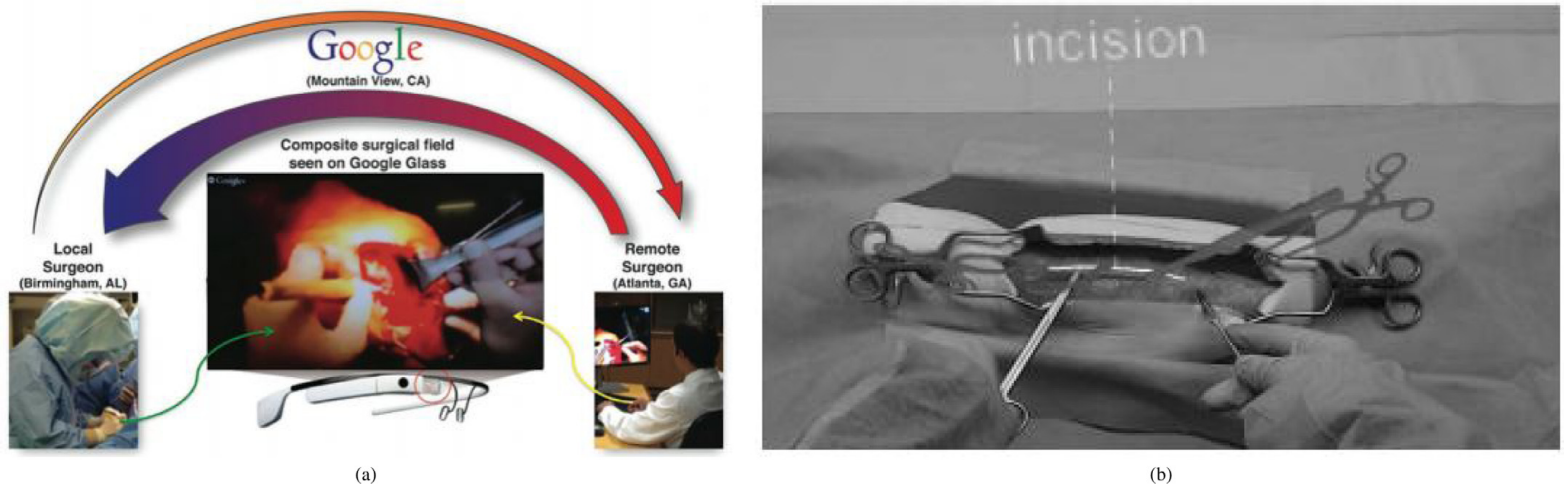
Telementoring Applications. (a) Overview of a Google Glass systeming using a composite surgical field *Source:*Fig. 3*of**Ponce et al. (2014)*. (b) First-person view of HoloLens-based holographic instructions consisting of 3D models and 3D lines *Source:*Fig. 2*of**Rojas-Muñoz et al. (2019)*.

Telementoring also belongs to the broader scope of surgical training, but involves a surgical trainee being mentored by an expert surgeon during a surgical procedure rather than training outside the operating room. Ponce et al. (2014) used a Google Glass based mentoring system for shoulder arthroplasty (Fig. 13(a)). The student surgeon and teacher surgeon can both see a composite surgical field in which hands and surgical tools of both surgeons can be seen at the same time. Rojas-Muñoz et al. (2019) used a HoloLens mentoring system in which an expert surgeon can place virtual 3D annotations (surgical tools and incision guidance lines) which are seen by the student surgeon in real time (Fig. 13(b)). The authors reported improved information exchange between student and mentor, reduced number of focus shifts and reduced placement error. A similar mentoring is presented in Rojas-Muñoz et al. (2020a), where trainees performed leg fasciotomies and reported an improved surgical confidence.

**Fig. 14:**
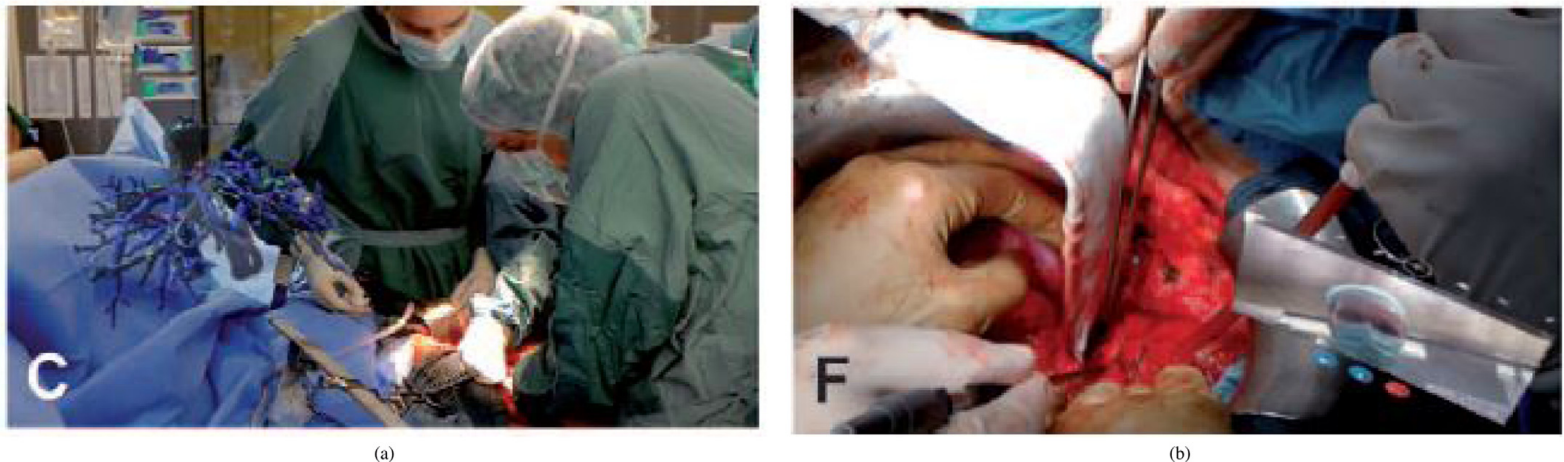
Surgical Anatomy Assessment and Teleconsultation Applications in Visceral Surgery: (a) Intraoperative visualisation of a preoperative model of the vascular anatomy of the cranio-ventral liver and tumor to be dissected. (b) Intraoperative tele-consulting: real-time video communication with a remote surgeon *(Source:*Fig. 3*(C and F) of**Sauer et al. (2017)*).

In contrast to telementoring, where the dialogue is continuous, teleconsultation (n=5) focuses on a consultation based on-demand communication between colleagues. Sauer et al. (2017) explored potential benefits of using the HoloLens to establish a web-service based real-time video and audio communication with a remote colleague during visceral-surgical interventions (Fig. 14). In addition, the remote surgeon could mark anatomical structures within the surgical site using a tablet computer. Borgmann et al. (2016) used a Google Glass for hands-free teleconsultation during different urological surgical procedures. Other examples use the Google Glass for consultation during reconstructive limb salvage (Armstrong et al., 2014) and orthopaedic procedures (Jalaliniya et al., 2017).

Applications where holographic 3D anatomy is displayed an intraoperative setup without trying to guide the surgical procedure are categorised as surgical anatomy assessment. This involves intraoperative assessment of preoperatively aquired patient anatomy that aids clinicial decision making without trying to guide the procedure itself.

Sauer et al. (2017) used a HoloLens based 3D visualisation of a liver cranio-ventral incl. tumor (Fig. 14(a)) to improve a surgeon’s spatial understanding of the target anatomy during dissection of the liver parenchyma in complex visceral-surgical interventions. Mitsuno et al. (2017) used Moverio BT-200 smart glasses and registered holographic 3D face and facial bones surfaces (Fig. 11(b)) to aid clinical decision making for more objective assessment of the improvement of a patient’s body surface contour in plastic surgery.

A further category of display shows preoperatively acquired 2D patient imaging data and medical records in the surgeon’s field of view using AR rather than a separate monitor. Borgmann et al. (2016) asked surgeons to rate their perceived usefulness of displaying patients’ medical records and CT scans on a Google Glass during urological surgical procedures. They found that reviewing patient images was rated less useful, whereas reviewing medical records received a high rating.  Jalaliniya and Pederson (2015) used a Google Glass to view and manipulate X-ray and MRI images.

## 6 AR visualisations

Conventional computer-assisted surgery uses different types of visualisations to aid preoperative planning or intraoperative procedures and a similar range of visualisations have been adopted for AR-assisted applications (see Table A.1).

**Fig. 15:**
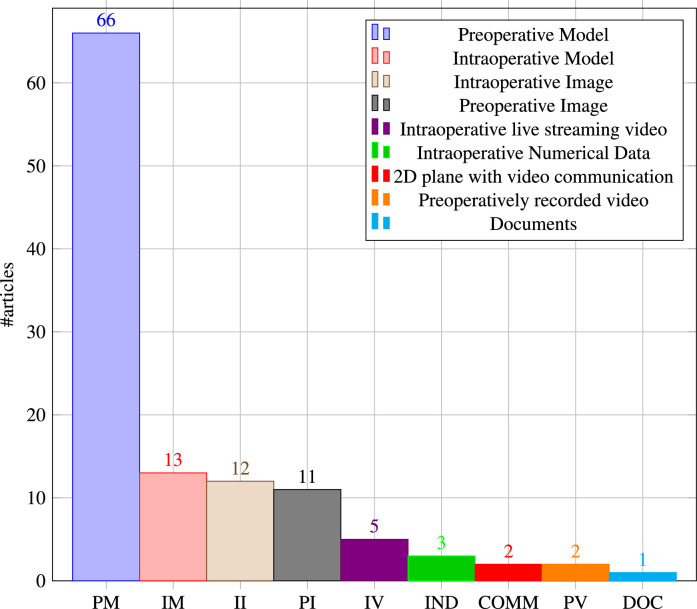
Distribution of included articles by **type of AR visualisation**

Fig. 15 shows the distribution of articles by type of AR visualisation. The majority of articles use preoperative models (n=66), usually consisting of 3D reconstructed patient anatomy generated from CT or MRI imaging content, sometimes in conjunction with preoperative planning components. Liebmann et al. (2019) used holographic preoperatively planned screw trajectories and drill entry points to aid pedicle screw placement in spinal fusion surgery. Pratt et al. (2018) investigated the usefulness of CT reconstructed 3D patient leg models including bony, vascular, skin and soft tissue structures, vascular perforators and a surrounding bounding box that facilitated manual registration.

We also consider non-anatomical content as a preoperative model such as holographic user interaction menus or graphical annotations. Rojas-Muñoz et al. (2020a), for example, used graphical annotations of incision lines and a model of surgical tools in a telementoring system. Condino et al. (2018) implemented a virtual menu with toggle buttons for a hybrid simulator for orthopaedic open surgery training.

Applications where 3D visualisations are generated intraoperatively in order to take updated live information into account, usually for surgical guidance, we refer to as intraoperative model visualisation (n=13). Katić et al. (2015) used live drill trajectory guidance information such as position and depth of dental drill and injury avoidance warnings in dental implant surgery. Lin et al. (2018) investigated utility aspects of intraoperatively generated needle visualisations such as needle position, orientation, shape and a tangential ray during needle biopsy.

Live intraoperative images (n=12) can be displayed in a surgeon’s field of view using AR in order to have crucial patient data available without the need to look at a separate monitor. Deib et al. (2018) displayed radiographic images to aid percutaneous vertebroplasty, kyphoplasty and discectomy procedures. Qian et al. (2018) used an endoscopy visualisation in the form of a 3D plane with video streaming content that aimed to increase the first assistant’s task performance in robot-assisted laparoscopic surgery. Fotouhi et al. (2019a) explored potential benefits of holgraphic C-arm interventional X-ray images registered to the C-arm view frustrum for guided k-wire placement in fracture care surgery.

The standard method of viewing preoperative images on a separate monitor away from the surgical site is often cited as a reason for pursuing AR guidance. Holographic visualisation of preoperative images (n=5) was proposed to allow visualisation on or near the surgical site. Song et al. (2018) incorporated 2D radiographic images with guidance information in their HoloLens-based endodontic treatment approach. Rynio et al. (2019) used 2D images with volume rendering, arterial diameters and planning notes to support endovascular aortic repair.

**Fig. 16:**
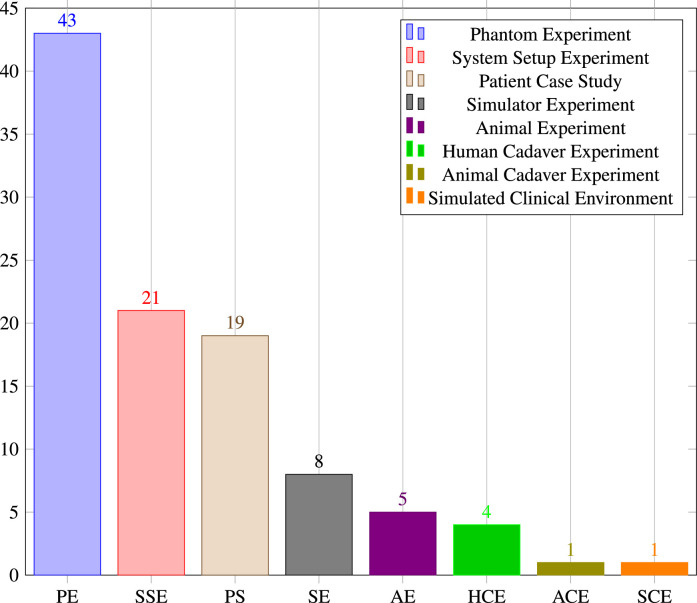
Experimental setting, from phantom to animal to clinical studies. Phantom studies dominate and though a number of clinical case studies have been reported (19), we are some way from proving clinical effectiveness of OST-HMDs at present.

The remaining categories of AR visualisations we identified in this review have only a few applications. Intraoperative live video streaming (n=5) is mostly used in telementoring applications. Ponce et al. (2014) used a hybrid image approach in which the mentee’s surgical field is combined with the hands of the remote expert surgeon. Dickey et al. (2016) presented an application in which an interactive video display is visible to the mentee that shows a cursor moved by the supervising physician. Intraoperative numerical data (n=3) is usually displayed as a 2D plane containing numerical data that aid clinical decision making or surgical guidance. Pietruski et al. (2019) displayed a cutting guide deviation coordinate system supporting a surgeon during mandibular resection. Schlosser et al. (2019) implemented a patient monitoring application comprising a holographic 2D screen that shows patient heart rate, blood pressure, blood oxygen saturation and alarm notifications. Another AR visualisation category uses a 2D plane with video communication software (n=2) and has been applied in reconstructive limb salvage procedures (Armstrong et al., 2014) and orthopaedic procedures (Jalaliniya et al., 2017). Preoperatively recorded video (n=2) was explored by Dickey et al. (2016) as a video guide during surgical training. Armstrong et al. (2014) used holographic visualisation of documents (n=1), with articles from a senior author being displayed in the surgical field of view (Fig. 16).

## 7 Validation of AR

All papers included in this review perform some kind of experiments to verify usability and the associated potential utility of their proposed OST-HMD assisted surgery solution. In this section we analyze the conducted experiments including a categorisation into an either quantitative or qualitative evaluation. An overview can be found in in the *Experiments* column of Table A.1.

### 7.1 Experimental setting

Phantom experiments dominate the list of papers (n=43). Phantoms may be stylistic or try to mimic anatomical correct structures and are either self-made, 3D printed or acquired from specialised companies. Researchers can test their developed methods on phantoms without involving real human or animal anatomy. Chen et al. (2015) used a 3D-printed cranio-maxillofacial model to verify the registration accuracy of their presented surgical navigation system, and a 3D pelvis model to test their navigation system. Deib et al. (2018) incorporated a lumbar spine phantom into the validation of their presented application for image guided percutaneous spine procedures. A guidance approach for pedicle screw placement, developed by Gibby et al. (2019), was tested using a phantom consisting of L1-L3 vertebrae in opaque silicone that mimics tissue properties.

System setup experiments (n=21) don’t use realistic target anatomy structures but verify the system’s intrinsic characteristics by conducting accuracy experiments in specific areas, such as system registration and calibration. Andress et al. (2018), for example, test the calibration step of their presented OST-assited fluoroscopic x-ray guidance system that uses a multimodal fiducial. The calibration experiment consists only of a HoloLens, a C-arm and a multimodality marker. Fotouhi et al. (2019a) conducted a similar experiment incorporating a hand-eye calibration experiment including a HoloLens, a C-arm and an optical tracker in their system that provides spatially aware surgical data visualisation. In order to verify the calibration accuracy of their proposed online calibration method for the HoloLens, Guo et al. (2019) used a calibration box with visual markers, a tracking device based on computer vision.

Patient case studies (n=19) present surgical procedures that were tested on one or more patients. Yoon et al. (2017) validated their OST-assisted spine instrumentation approach in which neuronavigation images were streamed onto a Google Glass on 10 patients. Mitsuno et al. (2017) tested their intraoperative body surface improvement approach on 8 patients, each with a different diagnosis. These clinical evaluations are very useful, but further studies will be required to establish clinical effectiveness and to demonstrate improved patient outcome.

The remaining five types of experiments that have been identified in this review have a comparatively small number of associated articles. Simulator experiments (n=8) take advantage of available simulation hardware allowing researchers or surgeons to mimic specific surgical procedures. Mahmood et al. (2018) used a physical simulator model (Fig. 12, 5.2) that allows users wearing a HoloLens to simulate a transesophageal echocardiography (TEE) examination.

Animal experiments (n=5) involve living animals that are anaesthetised and enable surgeons to test surgical applications under realistic conditions that consider physiological aspects such as respiratory motion. Zhou et al. (2019b) and Zhou et al. (2020) tested their surgical navigation system for LDR brachytherapy on a live porcine model (Fig. 9(c), section 5). Li et al. (2019) performed a similar in vivo test of their respiratory liver tumor puncture navigation system that takes respiratory liver motion into account. An animal cadaver experiment (n=1) was also performed by Katić et al. (2015), who used a pig cadaver to test their application for intraoperative guidance in dental implant surgery.

Human cadaver experiments (n=4) have the inherent benefit of allowing surgeons to test novel surgical procedures on real anatomic structures without risk to patients. Wang et al. (2016), for example, used six frozen cadavers with intact pelvises to investigate a novel method for insertion of percutaneous sacroiliac screws.

Finally, Jalaliniya et al. (2017) proposed a simulated clinical environment (n=1) to test clinical infrastructure elements and workflows rather than surgical procedures. A Google Glass based wearable personal assistant that allows surgeons to use a videconferencing application, visualise patient records and enables touchless interaction with preoperative X-ray and MRI images displayed on a separate screen without the need to use mouse or keyboard. The application was tested in different clinical setups comprising a simulation doll, human actors and real surgeons and nurses.

### 7.2 Evaluation methods

Evaluations may be quantitative experiments that collect measurable data such as registration accuracy or qualitative experiments gather descriptive information such as surgeons’ observations or opinions that cannot be measured. Most of the articles in this review contain some sort of quantitative experiments (n=88), whereas qualitative experiments have much fewer associated articles (n=11).

Quantitative experiments include registration accuracy evaluation (Chen, Xu, Wang, Wang, Wang, Zeng, Wang, Egger, 2015, Condino, Turini, Parchi, Viglialoro, Piolanti, Gesi, Ferrari, Ferrari, 2018, Gibby, Swenson, Cvetko, Rao, Javan, 2019), calibration accuracy evaluation (Andress, M. D., Unberath, Winkler, Yu, Fotouhi, M. D., M. D., Navab, 2018, Fotouhi, Unberath, Song, Gu, Johnson, Osgood, Armand, Navab, 2019, Qian, Deguet, Kazanzides, 2018) or intraoperative guidance verification such as tool positioning (Stewart and Billinghurst, 2016) or guide wire placement (Liebmann et al., 2019). Experiments in which a user has to give specific survey-based feedback is also classed as quantitative where the survey is predetermined and can be evaluated on a numerical basis.

Qualitative experiments are usually questionnaire based in which the participants detail specific observations that cannot be evaluated numerically. Deib et al. (2018), for example, designed an experiment in which the user had to complete a questionnaire following a surgical image-guided spine procedure describing benefits, limitations and personal preferences.

## 8 Registration and tracking in surgical AR

Whenever holographic anatomy visualisations need to be superimposed on respective patient anatomy, the question of registration accuracy arises. Registration refers to the establishment of a spatial alignment between the coordinate system of the patient space and the digital image space (Liu et al., 2017). In the context of AR-guided surgery it can be defined as achieving correspondence between superimposed visualisation and patient anatomy. Devices such as the HoloLens define their own coordinate system for the room and the user’s head is tracked within this space. The registration process places the preoperative model in HoloLens coordinates. If an external tracking system is used a further alignment between the devices is required. Tracking and registration each have potential errors and should be considered separately.

In most cases a rigid coordinate system transformation involving translation and rotation is optimised given some corresponding features (Wyawahare et al., 2009). The required accuracy of the established registration depends on the application. For OST-HMD AR-guided procedures deviations between visualisation and true target anatomy may lead to surgical errors resulting from misinterpreted spatial relationships. For OST-HMD AR, overall accuracy also depends on the user’s perceptual accuracy.

Appendix Table A.1 lists the main reported accuracy results of all included articles and the associated type of conducted experiment or experiments. Because of wide variation in experimental setup and different accuracy metrics used in the literature, direct comparison of articles based on the reported accuracy is difficult. Some articles report specific registration accuracy experiments (Chen, Xu, Wang, Wang, Wang, Zeng, Wang, Egger, 2015, Gibby, Swenson, Cvetko, Rao, Javan, 2019, Li, Si, Liao, Wang, Klein, Heng, 2019, Nguyen, Cardinell, Ramjist, Lai, Dobashi, Guha, Androutsos, Yang, 2020, Heinrich, Schwenderling, Becker, Skalej, Hansen, 2019), while others report the accuracy of specific experimental guidance task results that result from a preceding registration (Wang, Wang, Leong, Xu, Chen, Wang, 2016, Stewart, Billinghurst, 2016, Lin, Siu, Bae, Cutkosky, Daniel, 2018, Hiranaka, Fujishiro, Hida, Shibata, Tsubosaka, Nakanishi, Okimura, Uemoto, 2017).

A number of papers consider manual alignment of the virtual model by the surgeon for registration. When matching corresponding features the most common methods are point-based landmark registration and surface registration Liu et al. (2017).

### 8.1 Manual alignment

Pratt et al. (2018) propose manual registration for extremity reconstruction using the HoloLens. Manual registration directly aligns the model to the HoloLens coordinate system, so no further tracking calculation is required. Also, since the alignment is achieved by the user’s and to their satisfaction, no correction for individual’s 3D perception is needed. Fotouhi et al. (2020) propose manual registration for virtual-to-real alignment of a robotic arm that uses two reflective AR displays (Fig. 10(b)). The reflective AR displays act as holographic mirrors that allow the first assistant to see the virtual robot arm from multiple perspectives and therefore act as a registration aid. Experiments showed that using the reflective AR displays improved the accuracy from 30.2±23.9 mm to 16.5±11.0 mm. Nguyen et al. (2020) compared three manual registration methods for neuronavigation using the HoloLens: tap to place, 3-point correspondence matching and keyboard control. The authors also presented a novel statistics based method allowing researchers to quantify registration accuracy for AR-assisted neuronavigation approaches. The keyboard method was found to be the most accurate (for detailed accuracy results see appendix Table A.1).

Frantz et al. (2018) presented a neuronavigation approach which is based on manual registration using fiducial markers. Users can manually register a holographic visualisation of a 3D reconstructed CT scan human skull model to its physical counterpart via the help of virtual axes (Fig. 19(a)). Registration accuracy was measured by both localisation accuracy (Fig. 19(b)) and perceived holographic drift (Fig. 19(c)). The mean perceived holographic drift of the manual registration was 4.39 ± 1.29 mm. Maintaining hologram registration via continuous tracking of a marker resulted in a lower perceived hologram drift of 1.4 ± 0.67 mm.

These manual methods may not be of sufficient accuracy to meet the clinical requirements for guidance of surgical dissection, but the ability to orient structures can improve spatial awareness and may be useful in broader surgical decision making.

### 8.2 Point-based registration

Point-based registration matches corresponding pairs of fiducial points from one coordinate system to another. External fiducial markers may be attached to specific patient anatomy, such as bony structures in orthopaedic surgery or the skull in neurosurgery. Alternatively existing anatomical landmarks may be used. The same virtual fiducial points are usually marked using an external tracking device and can also be displayed on the holographic 3D anatomy model. A common accuracy measure for point-based methods is the fiducial registration error (FRE), which is the residual error of the mismatch between pairs of corresponding points after alignment. A better metric with more clinical relevance is the target registration error (TRE) at the surgical target (Seginer, 2011).

**Fig. 17:**
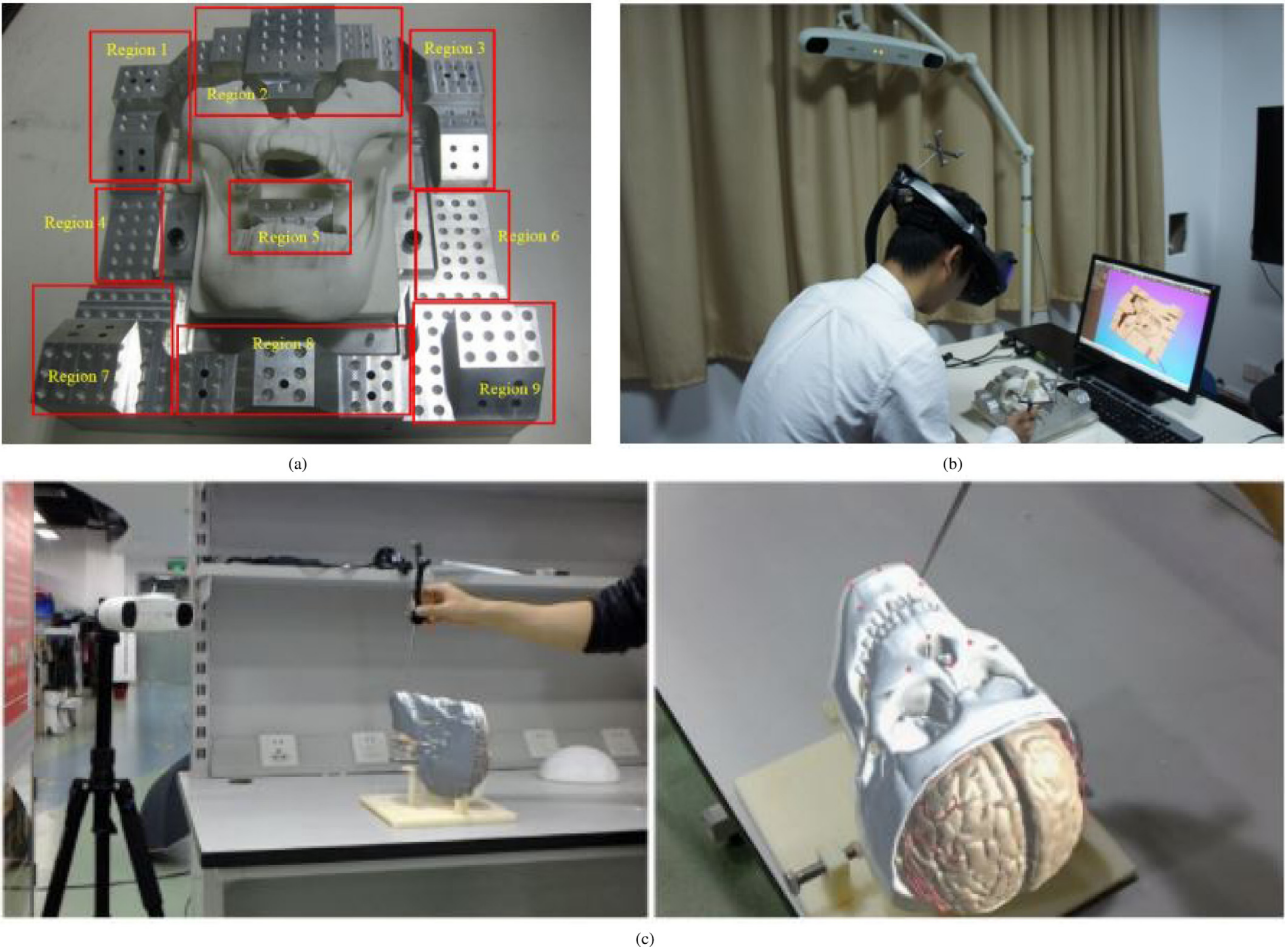
Accuracy verification experiment examples using optical trackers: (a) Accuracy verification block including a metal base with taper holes (for distance and angular error measuring) and 3D-printed cranio-maxillofacial model. (b) A user is conducting the accuracy verification experiment using the accuracy verification block, a tracked calibration tool and tracked OST-HMD *(source:*Chen et al. (2015)Fig. 7 and 8(c)). Registration accuracy validation using a 3D-printed skull with 10 landmarks (red dots) and a k-wire with attached optical marker *(source:*Li et al. (2019)Fig. 6).

Chen et al. (2015) perform point-based registration as an initial alignment before surface-based refinement (section 8.3) and conducted an accuracy experiment using a verification block (Fig. 17 (a)) using an optical tracking system with reflective markers Fig. 17(b)). The authors reported mean distance and angular errors of 0.809±0.05 mm and $1.038^{\circ }\pm 0.05^{\circ }$ respectively. Addressing the problem of incorrect needle placement and associated failed tumor ablation, Li et al. (2019) proposed a manual registration method using a HoloLens with an optical tracker to superimpose 3D liver models on patients for liver tumor puncture navigation. Optical markers rigidly attached to the HoloLens, anatomical marks on the patient and a k-wire with attached reflective spheres serving as an optical marker are used for an initial manual registration step. The tracked k-wire is then used for automatic temporal registration during the procedure. The authors performed a registration accuracy validation experiment using a 3D-printed skull with 10 landmarks (Fig. 17(c)) and reported an average target registration error of 2.24 mm.

**Fig. 18:**
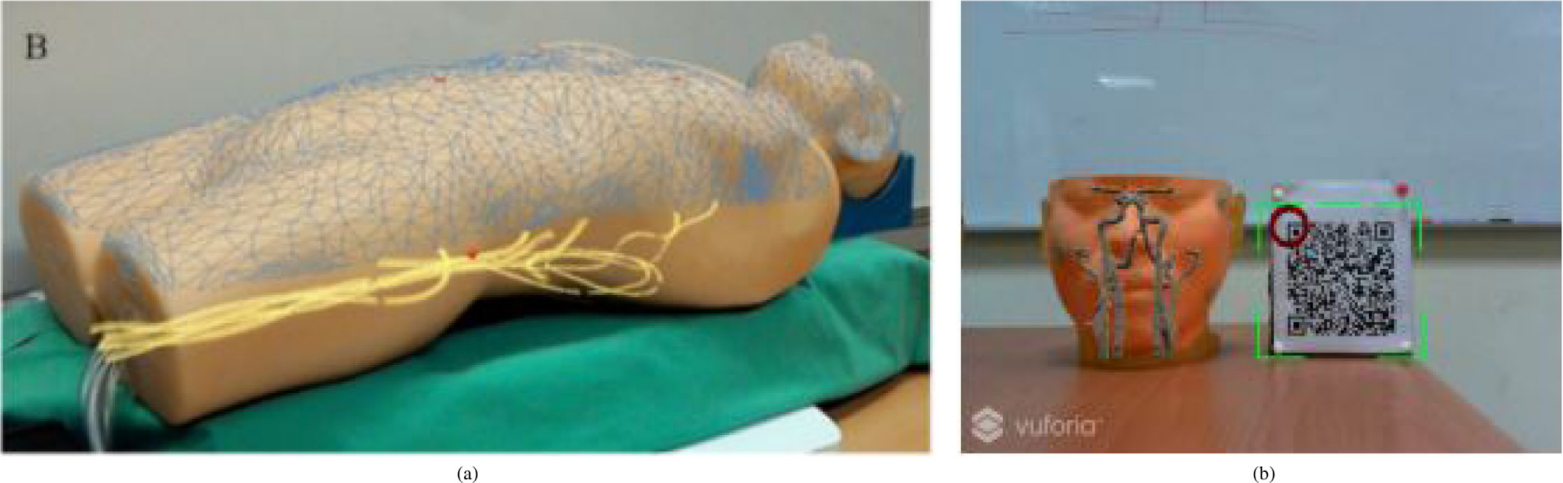
(a) Point-based registration: Human body surface mesh including vessel tree registered to a phantom by landmarks, where surface registration to the HoloLens surface failed *(source:*Kuhlemann et al. (2017)Fig. 1). (b) Surface registration result: Dummy Head with superimposed 3D CT scan reconstruction of head and intracranial vasculature. HoloLens camera detection of the QR code provides tracking *(source:*Wu et al. (2018), part of Fig. 9b).

Another point-based registration approach for catheter navigation was presented by Kuhlemann et al. (2017) and tested on a human body phantom (Fig. 18(a)): A CT reconstructed 3D body surface mesh including marching cubes segmentation of a vessel tree was registered to a body phantom using landmarks with a reported accuracy of 4.34±0.709 mm (FRE).

### 8.3 Surface registration

Point-based registration is an alignment process that matches anatomical or fiducial landmarks. Surface registration offers the possibility of alignment without specific fiducial markers. Using a laser range scanner or a tracked probe, a point cloud is collected from the surface of the patient’s target anatomy (e.g. the head) (Liu et al., 2017). Another surface or point cloud is derived from the image space and an algorithm is then used to match both point clouds. Most surface registration methods require a coarse manual or point-based registration step to place the image-based point cloud must be placed close to the target registration pose before the algorithm proceeds. Iterative closest point (ICP) is a popular realisation of a surface based registration and has been applied in several of our selected articles.

The HoloLens internal tracking method produces a generated surface mesh and Kuhlemann et al. (2017) investigated whether this could be used for surface registration. A CT scan derived body surface was matched to the HoloLens surface mesh. But the HoloLens mesh resolution was found to be too coarse. In addition, Frantz et al. (2018) also reported that the HoloLens’ built-in spatial mesh and simultaneous localisation and mapping (SLAM) system is unsuitable for registration and subsequent tracking due to the low vertex density and surface bias of the generated mesh and uncertainty in the SLAM realisation. Wu et al. (2018) presented an improved version of the ICP algorithm for medical image alignment that aims to provide a global optimum via a stochastic perturbation. A dummy head alignment test revealed an average target registration error of <3 mm. Fig. 18 (b) shows an example registration result.

### 8.4 Other registration methods

Other types of registration have also been explored. Liu et al. (2019) applied a Fourier transformation based registration method in their intraoperative guidance approach for structural heart disease for transcatheter procedures. The authors used a 3D reconstructed spine image and a segmented spine from an intraoperative fluoroscopy to calculate a Fourier-based scale and rotational shift which was then used to register the fluoroscopic image to the respective 3D model of the spine. The Fourier based registration achieved an accuracy of 0.42±0.02mm.

A HoloLens specific marker-less automatic registration method for maxillofacial surgery is presented by Pepe et al. (2019). Their algorithm accesses the HoloLens’ built-in RGB camera and extracts facial landmarks from the camera’s video stream. Via known virtual-to-real world transformations of the landmarks and spatial mapping information from the HoloLens’ Spatial Mapping API, the algorithm then computes the registration. The achieved average positioning error of the x, y, z axes was 3.3±2.3 mm   y: -4.5±2.9 mm and z: -9.3±6.1 mm respectively.

**Fig. 19:**
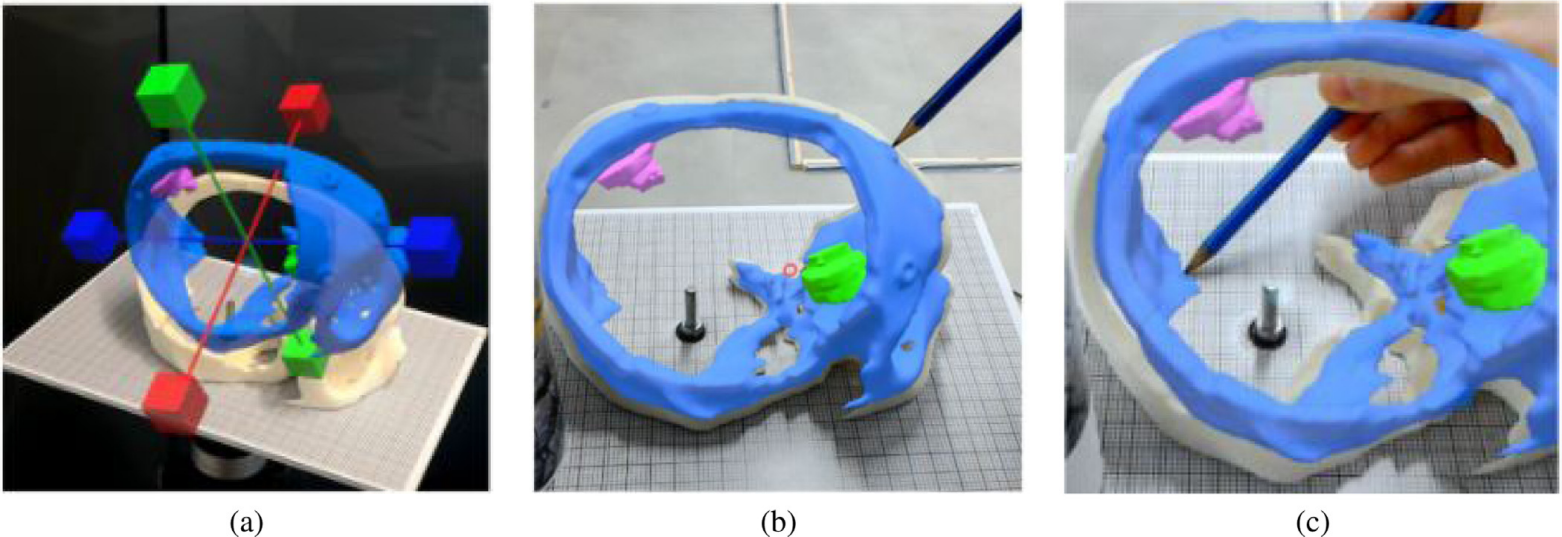
Registration accuracy verification using a sheet of millimeter paper: (a) Manual and point-based registration: Virtual axes allow the user to translate and rotate a human skull model in order to align it with a phantom. Fiducial markers serving as registration aids are present on both the virtual model and the phantom. (b) Localisation accuracy measurement is realised by placing the tip of a stylus into the center of a holographic fiducial marker. (c) By calculating the difference in similar points the perceived hologram drift is measured. *(source:*Frantz et al. (2018)Fig. 4a, 4b and 5a).

### 8.5 Tracking

Having established a registration, any subsequent motion of either the patient or the surgeon must be tracked to maintain the alignment. A summary of tracking methods is given in Table 3.

**Table 3:** Papers by tracking method.

Tracking method totals		Tracking marker totals	
**External tracker**	19		
NDI Polaris	11	Reflective spheres	14
NDI EM/Aurora	4	EM	4
OptiTrack	1		
PST Base	1		
VICON	1		
Custom webcam tracker	1	Coloured catheter segments	1
**Tracking with HMD camera**	20	**Optical Markers**	18
HoloLens	17	AprilTag	1
Other	3	Aruco	1
		ARToolkit	3
		Custom	4
		Vuforia	9
**Markerless tracking**	11		

#### 8.5.1 Markerless tracking

The HoloLens inherently tracks the surgeon’s head and providing the patient position is fixed within the operating room, this may be a sufficient method in itself. Eleven papers use only the HoloLens tracking and these are associated with the manual registration process described in Section 8.1. The advantage of this method is that no external measurement device is required and no markers need to be physically attached to the patient, hence the name markerless tracking. This can be a significant advantage in terms of sterility, convenience and operative workflow integration. However, the accuracy is user dependent and may not be sufficient for some surgical tasks. Pratt et al. (2018) and Scherl et al. (2020) use manual alignment to the anatomy, whereas Creighton et al. (2020) register to fiducial markers for guidance of targets in the skull base.

#### 8.5.2 Tracking of markers using the OST-HMD device

OST-HMD devices such as the HoloLens incorporate cameras into their tracking process. These cameras can be used to track surface features or markers placed in the surgical field, accounting for 20 of our papers. It is common for these markers to be small planar identifiable markers modelled on QR codes. Several quite similar free libraries are available for this purpose, including Aruco, ARToolkit and AprilTags. Andress et al. (2018) use ARToolkit markers that are also visible in X-ray to align to fluoroscopic views for orthopaedics. Liebmann et al. (2019) use the stereo HoloLens camera sensors in research mode to track planar sterile markers for pedicle screw navigation. Some authors use their own custom markers, such as the cube and hexagonal markers used by Zhou et al. (2019b) in their system for brachytherapy. The commercial Vuforia package can also be used to track any planar printed image and accounts for half of the marker-based tracking through the OST-HMD (9 papers).

One advantage is that the OST-HMD camera’s position is relative to the surgeon, so no extra registration is needed and the direction of the camera shoud be towards the surgical field. While this can be effective, the resolution and field of view of the cameras may not be best designed for tracking within the surgical target area.

#### 8.5.3 External tracking devices

There are several commercially available devices that are able to track markers within the operating room. It is clear from table 3 that Northern Digital Inc. (NDI) dominate this field, with the Polaris optical tracker accounting for 11 papers and their electromagnetic tracker, Aurora, a further four papers. Li et al. (2019) use the Polaris for liver biopsy in the presence of breathing, whereas Kuhlemann et al. (2017) use EM tracking for endovascular interventions. Other system are optical and account for one paper each (OptiTrack, PST Base and VICON). Apart from one custom tracker based on a webcam for catheter tracking (Sun et al., 2020b) all optical systems use passive reflective spherical markers.

It may be invasive to attach such markers rigidly to the patient, but such methods form part of several commercial image guidance systems and this is probably the most accurate way to achieve and maintain alignment.

## 9 Human factors

OST-HMDs are wearable technological devices that enable the user to visualise and/or interact with 3D virtual objects placed within their normal view of the world. These unfamiliar devices present a novel form of human-computer interaction (HCI) and their acceptability by surgeons will depend on HCI factors. Technological aspects, such as the size of the augmented field of view or system lag during streaming of video content, can affect user acceptance. But beyond these are human factors that may vary from user to user but are crucial to the utility of a technological interaction device. They encompass perceptual, cognitive and sensory-motor aspects of human behavior that drive the design of HCI interfaces to optimise operator performance Papantoniou et al. (2016).

However, attempts to identify consistent generic human factors that capture basic human behavior and cognition that apply to the design of optical HCI systems has been problematic and HCI design guidelines incorporating consistent human factors have not yet been established. When addressing the negative side effects of HCI aspects only, human factors are sometimes considered as human limitations. Highlighting the aspect of human error, Lowndes and Hallbeck (2014) addressed aspects of human factors and ergonomics in the operating room in general with a focus on MIS and found that most medical errors are a result of suboptimal system design causing predicable human mistakes. They also state that despite efforts made by human factors and ergonomics professionals to improve safety in the operating room for over a century, increasingly complex surgical procedures and advances in technology mean that consideration of human interaction will be required to help users cope with increasing information content. We believe that similar safety aspects of human factors also apply in OST-HMD assisted surgical applications.

### 9.1 Human factors in AR

In more general non-surgical AR applications, human factors have played an important role and have been explored in the context of HCI. Livingston (2005) evaluated human factors in AR in 2005 and found that apart from technological limitations, human factors are a major hurdle when it comes to translation of AR applications from laboratory prototypes into commercial products. To determine the effectiveness of AR systems requires usability verification, which led them to the following two research questions: *1.) How to determine the AR user’s key perceptual needs and the best methods of meeting them via an AR interface? 2.) Which cognitive tasks can be solved better with AR methods than with conventional methods?* They attempt to a solution for these two questions by conducting limited but well-designed tests aiming to provide insights into HCI-design aspects that lead to utility for perceptive and cognitive tasks. These consist of low-level perceptual tests of specific designed visualisations on the one hand and task-based tests that focus only on the well-designed part of the user interface. In Huang et al. (2012), Livingston points out that designing cognitive tasks for usability evaluation seems to be easier than designing low-level perceptual tasks, since cognitive tasks naturally arise from the given AR application, whereas it is rather challenging to design general low-level perceptual tasks that have wider applicability. He also states that the design of a perceptual task determines how generalisable the evaluation results are beyond the specific experimental scenario and indicates that a solution may be to design general perceptual tasks that verify the usability of hardware. Finding general perceptual tasks is not always easy when hardware limitations interfere with the task design. If the effect of a hardware related feature influences a user’s cognition on top of his/her perception, the dependence on the perceptual task will be increased as well. An example for such an effect is system latency in a tracking device.

### 9.2 HCI design considerations in OST-assisted surgery

Though human factors in the context of HCI design considerations in OST-HMD assisted surgery are likely to be very important to the success of any system, only a few examples of such research can be found in the literature. In addition to the need for careful experimental design that allows a generalised result, there are also technical aspects that can be addressed to minimise unwanted human behavior when using OST-HMDs. Such technical aspects were explored by Tang et al. (2003), who evaluated human factors in variants of the *Single point active alignment method (SPAAM)* for OST-HMD calibration that require human-computer interaction. They aimed to answer the question why calibration of OST-HMDs is challenging for users; and found that human factors have a major impact on calibration error and therefore lead to significantly different accuracy results for different users. They proposed the following guidelines for the design of OST-HMD calibration procedures: *1.) Calibration should not rely on head movements only, 2.) The user’s head should be kept stabilised by minimizing extrinsic body movements* and *3.) Careful consideration of the data collection sequence for the left and right eye so that calibration error does not bias towards a dominant eye.*

Guo et al. (2019) also described the importance of human factors in the context of OST-HMD calibration. They proposed an online calibration method for the HoloLens and concluded that the accuracy of their calibration method is difficult to measure objectively since human factors impact the overall HCI experience as well as influencing the calibration accuracy.

The relationship between conscious and unconscious cognitive processes should be considered as well when considering the importance of human factors in HCI. Jalaliniya and Pederson (2015) addressed the necessity to consider an egocentric interaction when designing wearable HCI systems and replaced the terms *input* and *output* with *action* and *perception*. According to the authors, an improved understanding of a human’s perception, cognition and actions are necessary prerequisite when it comes to the design of a HCI system that offers better cognitive support.

### 9.3 Human factors identification

We identified numerous human factors in the 91 included articles. Even though the majority of articles didn’t use the term *human factors* explicitly, we included all user-related aspects described by the authors that have a potential impact on the acceptance, utility and performance of surgery with or without the proposed OST-HMD solution.

We identified 34 human factors that are described in Table 4. The human factors are grouped into two categories: 1.) Human factors of conventional surgery that are addressed by OST-HMD AR and 2.) Persistent human factors that remain or are an inherent part of the proposed OST-HMD solutions. We further categorize them into three phases of user interaction that are defined by the US Food and Drug Administration as part of a medical device user interface in an operational context (Health and Radiological, 2016): 1.) Information Perception (IP), where the information from the device is received by the user 2.) Cognitive Processing (CP), where the information is understood and interpreted and 3.) Control Actions (CA), where this interpretation leads to actions.

### 9.4 Human factors of conventional surgery that are addressed by OST-HMD AR

**Fig. 20:**
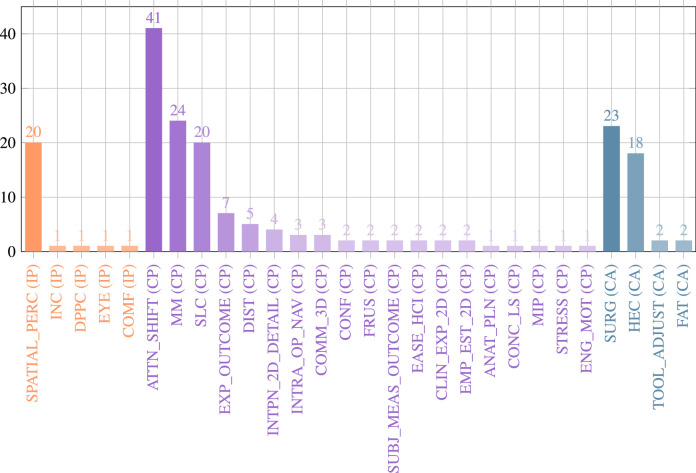
Distribution of human factors of conventional non-AR surgical approaches alleviated by the use of OST-HMD AR, grouped into the three categories 1. Information Perception (IP), 2. Cognitive Processing (CP), 3. Control Actions (CA)

Fig. 20 shows the distribution of all human factors (out of the identified 34 ones) described as being a limitation of conventional non-AR surgical methods that the authors aimed to address with their proposed OST-HMD solution. We group these into the categories IP, CP and CA and describe the most popular in more detail.

#### 9.4.1 Information perception related human factors of conventional surgery

*Spatial perception/awareness (SPATIAL_PERC):* A fundamental limitation of conventional image guidance methods appears to be the fact that crucial patient anatomy can only be perceived in 2D and hence prevents the surgeon from developing a personal sense of spatial perception and spatial awareness, which is a human factor we identified in (n=20) articles and hence the dominating human factor of conventional surgery in the IP category. Impaired spatial awareness has the unwanted side effect of an increased likelihood of surgical errors due to misinterpretation of anatomical spatial relationships. Qian et al. (2018) aim to increase a first assistant’s spatial awareness during robot-assisted laparoscopic surgery by providing a HoloLens solution in which a holographic endoscopy visualisation is registered within the personal viewing frustrum. Fotouhi et al. (2019a) addressed the problem of missing spatial context when looking at C-arm X-ray anatomy images on an external 2D monitor and presented a spatially aware HoloLens visualisation in which X-ray images are displayed in the correct spatial position of the patient’s anatomy with a surgeon’s view frustrum.

#### 9.4.2 Cognitive processing related human factors of conventional surgery

*Attention switch between surgical site and separate computer monitor (ATTN_SHIFT):* The human factor with the highest number of articles (n=41) in the CP category (and the dominating factor accross all three categories IP, CP and CA) is the *attention switch between the surgical site and a separate computer monitor*. In computer-assisted surgery a surgeon has to look away from the surgical site in order to see patient anatomy or surgical navigation information, and even switch between the surgical site and the screen multiple times during an operation. This inability to see both the surgical site and important patient anatomy or guidance information at the same time causes unwanted human behavior such as inconvenience and may also impact the continuity of the surgery Chen et al. (2015). Especially during image-based surgical navigation, the surgeon has to constantly switch his attention while manipulating surgical navigation tools which comes with unwanted side effect such as unfamiliar hand-eye coordination, distraction and loss of concentration Wang et al. (2016).

*Mental Mapping of 2D image data to the 3D World (MM):* An inherent problem of conventional computer-assisted surgery is that patient imaging data and surgical navigation information is displayed in 2D. This leads to the fact that the surgeon has to mentally map (or project) 2D image data onto the 3D world in order to translate the information seen on the 2D screen to the patient or surgical navigation tool. We identified *mental mapping of 2D image data to the 3D world* in (n=24) articles. Andress et al. (2018), for example, addressed the problem of mental mapping in the context of intraoperative guidance in percutaneous orthopaedic surgical procedures in which a surgeon has to place tools or implants precisely under C-arm based fluoroscopic imaging. The mental projection is counterintuitive and error-prone as a result of high mental workload and mental projective simplification.

*Steep learning curve (SLC):* Some conventional surgical procedures, especially those related to image guidance, require surgeons to overcome a *Steep Learning Curve* (n=20) due to the method’s inherent complexity. Lin et al. (2018) address this problem in needle guidance procedures that require considerable learning efforts due to the fact that physicians have to recover 3D information from 2D images, that the needle may cause artifacts in the images which hinder correct identification of needle tip and target and that complex hand-eye coordination is required to register the 2D images seen on a separate monitor to the patient anatomy Lin et al. (2018). Their OST-HMD AR system aims to reduce this learning curve.

#### 9.4.3 Control action related human factors of conventional surgery

*Increased risk of surgical error (SURG):* Several researchers addressed the risk of surgical error (n=23) which appears to be a common problem in some conventional image-guided procedures. El-Hariri et al. (2018) highlights the fact that conventional surgical navigation systems cannot observe the surgical scene and the external navigation computer monitor at the same time as being a potential problem that OST-HMD based solutions aim to solve. In another example, Song et al. (2018) aim to prevent or reduce errors in root canal treatments such as accidental perforation during access cavity creation.

*Unfamiliar hand-eye coordination (HEC):* The fact that a surgeon has to look away from the surgical site to a separate screen (see Section 9.4.2) while simultaneously manoeuvring surgical tools in image guided navigation causes unfamiliar hand-eye coordination because the surgeon cannot see his hands while looking on the separate screen Wang et al. (2016). *Unfamiliar hand-eye coordination* is tackled in (n=18) articles. Qian et al. (2018), for example, addressed a first assistant’s impaired hand-eye coordination during blind placement of robotic and hand-held instruments in conventional robot-assisted laparoscopic surgery by registering the holographic endoscopy visualisation with the visualised endoscope view frustrum (see Fig. 10 (a) of Section 5). Another example is given by Deib et al. (2018) who mention that a fundamental problem of conventional image guided percutaneous spine lies in an indirect guidance visualisation because radiography monitors showing fluoroscopic images are not aligned with the surgical site, which in turn hinders hand-eye coordination.

### 9.5 Persistent human factors of proposed OST-HMD solutions

Since OST-HMDs expose the user to new and possibly unfamiliar visual perception and interpretation as well as interaction options, the proposed OST-HMD solutions also introduce new human factors that should be taken into account when designing effective HCI. We refer to these as *persistent* human factors because they remain as issues of the proposed OST-HMD based solution. Table 21 shows the distribution of persistent human factors, some of which we discuss in the following sections. Analogous to section 9.4, the human factors are grouped into the categories IP, CP and CA and the most popular are detailed.

**Fig. 21:**
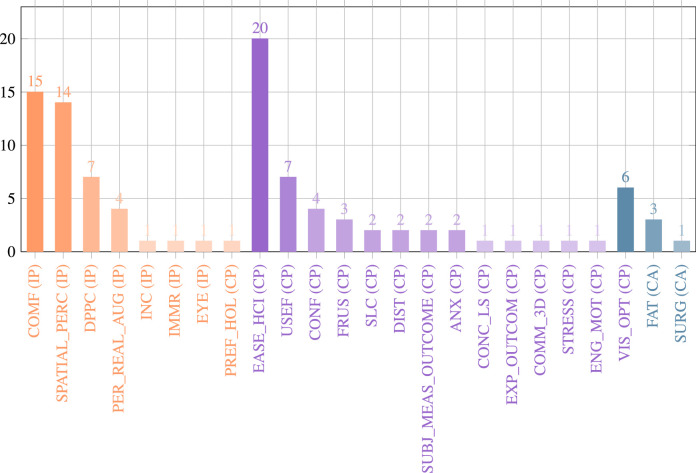
Distribution of persistent human factors of the proposed AR surgical approaches, grouped into the three categories 1. Information Perception (IP), 2. Cognitive Processing (CP), 3. Control Actions (CA)

#### 9.5.1 Information perception related human factors of ar-assisted surgery

*Perceived comfort level when wearing OST-HMD (COMF):* As is the case for all HCI devices including computers or laptops, one of the most important factors that influence user acceptance is the comfort level. Discomfort will inevitably prevent a device from becoming a routine instrument that users enjoy working with. *Perceived comfort level when wearing OST-HMD* was mentioned in (n=15) articles, and is therefore one of the human factors that dominate the IP category. Pietruski et al. (2019) presented a Movierio BT-200 Smart Glasses based intraoperative navigation system that supports mandibular resection and conducted a phantom experiment in which osteotomies were performed. Surgeons reported good long term wear work ergonomics. Rojas-Muñoz et al. (2020a) created a HoloLens telementoring system that allows surgeons to perform mentored leg fasciotomies. Participants reported that the weight of the HoloLens has a negative impact on their posture and comfort.

*Spatial perception/awareness (SPATIAL_PERC):* Spatial perception and spatial awareness was already described in Section 9.4 as a human factor of conventional non-AR methods, where 3D patient anatomy had to be inferred from 2D data. However, these spatial processing capabilities area also factors of 3D holographic visualisations and should also be taken into account for OST-HMD solutions, as reported in (n=14) articles. Given that AR exposes users to new perceptual stimuli that are usually not part of their normal experience, it is likely that users processes this new visual information differently, which in turn impacts the quality of the HCI during OST-HMD assisted surgical procedures. Condino et al. (2018) presented a HoloLens based hybrid simulator for orthopaedic open surgery that allows users to visualise 3D anatomy prior to performing a virtual viewfinder assisted surgical incision. Study participants who conducted a simulator experiment were engineers and clinicians. Results from a 5-point Likert questionnaire indicate that both user groups found it rather easy to perceive spatial relationships between real and virtual content; however, engineers tend to rate the ease of spatial relationship perception slightly higher than clinicians. Gnanasegaram et al. (2020) investigated in how far ear anatomy learning can be improved compared to conventional didactic lectures and computer modules. Study participants performed a spatial exploration of holographic ear models displayed on a HoloLens and rated the OST-HMD higher than didactic lectures and computer modules in terms of 1.) overall learning effectiveness, 2.) the learning platform’s ability to convey anatomic spatial relationships and 3.) learner engagement and motivation.

*Missing/impaired depth perception (DPPC):* Individual depth perception capabilities influence the ability to understand three dimensional holographic relationships as well as relationships between real and virtual objects. This may decrease the utility of systems that require perceptual precision. Several articles indicate missing or impaired depth perception as one of the limitations of the proposed OST-HMD approach (n=7). Andress et al. (2018) developed a fluoroscopic X-ray guidance system for percutaneous orthopaedic surgery that is based on a cocalibration of a C-arm to a HoloLens and aims to facilitate the perception of spatial relationships between patient anatomy and surgical tools. A phantom based K-wire insertion experiment revealed that the HoloLen’s build-in characteristic of rendering all holographic content at a focal distance of around 2m impacts the the user’s depth perception, and hence leads to an impaired interaction between real and virtual objects.

#### 9.5.2 Cognitive processing related human factors of AR-assisted surgery

*Perceived degree of ease and intuitiveness of HCI (EASE_HCI):* A fundamental aspect that plays a pivotal role when it comes to user acceptance of a proposed OST-HMD solution is the *perceived degree of ease and intuitiveness of HCI* which was the human factor with the most associated articles (n=20) in the CP category. Deib et al. (2018) presented a HoloLens based application for image guided percutaneous spine procedures that was tested in a phantom experiment in which percutaneous vertebroplasty, kyphoplasty and discectomy interventions were performed. Participants could select their preferred holographic visualisation mode and questionnaire results revealed that initially the most popular mode was the option that was closest to a conventional 2D monitor and hence the most intuitive one. However, after the user became familiar with the OST-HMD environment, the preferred mode of visualisation changed to one that offers more benefits of the new mixed reality environment. Jalaliniya et al. (2017) designed a Google Glass based personal assistant for surgeons for which users who conducted experiments had to complete a questionnaire which revealed that some users prefer hand gesture interaction over voice interaction because voice interferes with their patient communication.

*Perceived usefulness of OST-HMD (USEF):* Since OST-HMDs are not well established in operating theatres and part of routine surgical procedures clinicians can always compare OST-HMD solutions with conventional methods and thus decide for themselves whether the new AR approach is useful or not. It is therefore not surprising that *perceived usefulness* is a human factor mentioned in several articles (n=7). Borgmann et al. (2016) conducted a feasibility of Google Glass assisted urological procedures in which surgeons could access holographic preoperative CT scans. A patient case study with a five-point Likert scale evaluation involving 7 surgeons over 10 procedures totalling 31 procedures revealed that the system’s overall usefulness was rated as very high or high by 74% of the surgeons.

#### 9.5.3 Control action related human factors of AR-assisted surgery

*Selection of preferred mode of visualisation (VIS_OPT):* Sometimes users are given the possibility to optimize their HCI experience by selecting one out of several different modes of visualization. The *Selection of preferred mode of visualisation (VIS_OPT)* considers personal optimisation of the HCI (n=6) is the human factor with the most associated number of articles (n=6) in the CA category. An example is described in Qian et al. (2018): a first assistant has the option between two modes of endoscopy visualization- during robotic surgery: 1.) A holographic monitor capturing the endoscopy camera stream or 2.) an endoscopy visualization that is registered with the viewing frustrum (Fig. 10 (a)).

## 10 Discussion

In this review we summarise the current proposed applications of OST-HMDs in surgery. Orthopaedic surgery applications are the most popular (30.16%) and are mainly assisted intraoperative guidance applications, perhaps because it involves rigid bony structures and is a field where conventional guidance systems to achieve good implant alignment have become commonplace (Table 5).

**Table 5:** Addressed and persistent human factors (notation: human factor(s) on the left side of the arrow cause other human factor(s) on the right side of the arrow).

Study	Addressed Human Factors	Reported Persistent Human Factors
Lin et al. (2018)	MM, INTPN_2D_DETAIL, HEC, SLC	N/A
Qian et al. (2018)	ATTN_SHIFT → HEC, TOOL_ADJUST, DPPC, EXP_OUTCOME, SPATIAL_PERC	VIS_OPT
Chen et al. (2015)	ATTN_SHIFT → INC	N/A
Wang et al. (2016)	[ATTN_SHIFT → HEC, DIST, CONC_LS], SLC	N/A
Deib et al. (2018)	ATTN_SHIFT → HEC	EASE_HCI, SLC, VIS_OPT, COMF
Andress et al. (2018)	ATTN_SHIFT → DIST, MM, SLC	DPPC
Condino et al. (2018)	SUBJ_MEAS_OUTCOME, SLC	PER_REAL_AUG, FAT, IMMR, EASE_HCI, SPATIAL_PERC
Stewart and Billinghurst (2016)	ATTN_SHIFT → DIST	EYE
Gibby et al. (2019)	ATTN_SHIFT → HEC	VIS_OPT
Yoon et al. (2017)	ATTN_SHIFT, HEC	CONC_LS, ANX
de Oliveira et al. (2019)	ATTN_SHIFT, SLC	N/A
Aaskov et al. (2019)	[INTPN_2D_DETAIL → SURG]	PER_REAL_AUG
Liebmann et al. (2019)	INTRA_OP_NAV	N/A
El-Hariri et al. (2018)	[ATTN_SHIFT → SURG_ERR], [MM → SLC]	N/A, DPPC
Fotouhi et al. (2019a)	[ATTN_SHIFT → HEC], [MM → FRUS], SPATIAL_PERC	N/A
Meulstee et al. (2019)	ATTN_SHIFT, MM	N/A
Song et al. (2018)	[ATTN_SHIFT → SLC & SURG_ERR], DPPC	N/A
Mitsuno et al. (2017)	ATTN_SHIFT, SUBJ_MEAS_OUTCOME	PREF_HOL, PER_REAL_AUG
Pratt et al. (2018)	[INTPN_2D_DETAIL → SURG_ERR], DPPC	N/A
Brun et al. (2019)	DPPC, COMM_3D, MM	EASE_HCI
Li et al. (2017)	EMP_EST_2D, EASE_HCI, CLIN_EXP_2D	EASE_HCI
Zou et al. (2017)	EMP_EST_2D, EASE_HCI	EASE_HCI
Liu et al. (2019)	[DPPC, INTPN_2D_DETAIL → INTRA_OP_NAV]	EASE_HCI, VIS_OPT
Pietruski et al. (2019)	[ATTN_SHIFT → HEC], DPPC, SPATIAL_PERC	COMF
Kaneko et al. (2016)	[ATTN_SHIFT → HEC]	N/A
Kuhlemann et al. (2017)	MM	EASE_HCI
Karmonik et al. (2018)	COMM_3D	EASE_HCI
Frantz et al. (2018)	MM, ATTN_SHIFT	SPATIAL_PERC
Fotouhi et al. (2020)	SPATIAL_PERC, TOOL_ADJUST, SLC	PER_REAL_AUG
Hiranaka et al. (2017)	[ATTN_SHIFT → SURG_ERR]	N/A
Katić et al. (2015)	STRESS, MIP, [ATTN_SHIFT → ERG, SURG_ERR]	VIS_OPT
Borgmann et al. (2016)	N/A	USEF
Unberath et al. (2018)	MM, INTRA_OP_NAV	SPATIAL_PERC, SUBJ_MEAS_OUTCOME
Sauer et al. (2017)	MM, [ATTN_SHIFT → HEC], SPATIAL_PERC	COMM_3D
Armstrong et al. (2014)	SLC	N/A
Mahmood et al. (2018)	SLC, MM, SPATIAL_PERC	SPATIAL_PERC, FAT
Rojas-Muñoz et al. (2019)	ATTN_SHIFT, MM, FRUS, DPPC	FRUS
Rojas-Muñoz et al. (2020a)	CLIN_EXP_2D, SLC, HEC, EXP_OUTCOME	ANX, COMF, CONF
Pelanis et al. (2020)	MM, SPATIAL_PERC	SPATIAL_PERC, COMF
Nguyen et al. (2020)	[ATTN_SHIFT → SURG]	N/A
Zhou et al. (2019b)	MM, SLC	N/A
Pietruski et al. (2020)	MM, ATTN_SHIFT	N/A
Chien et al. (2019)	ATTN_SHIFT	N/A
Zhang et al. (2019)	[ATTN_SHIFT → MM, HEC], SPATIAL_PERC	COMF
Heinrich et al. (2019)	ATTN_SHIFT, MM	DPPC
Zhou et al. (2020)	ATTN_SHIFT, SURG	SLC
Wellens et al. (2019)	[ANAT_PLN → SURG]	SPATIAL_PERC
Fotouhi et al. (2019b)	MM	SPATIAL_PERC
Baum et al. (2020)	MM, EXP_OUTCOME, SLC, SPATIAL_PERC	SPATIAL_PERC
Liounakos et al. (2020)	[ATTN_SHIFT → HEC]	COMF
Jalaliniya et al. (2017)	ATTN_SHIFT	EASE_HCI
Rynio et al. (2019)	SPATIAL_PERC	N/A
Boillat et al. (2019)	SURG	N/A
Zhou et al. (2019a)	SURG, ATTN_SHIFT, HEC	DPPC
Schlosser et al. (2019)	DIST, FAT	DIST
Ponce et al. (2014)	SLC	COMF
Guo et al. (2019)	EYE, SUBJ_MEAS_OUTCOME	SUBJ_MEAS_OUTCOME
Dickey et al. (2016)	SLC, DIST	DIST, USEF, EASE_HCI
Al Janabi et al. (2020)	[ATTN_SHIFT, HEC → SURG, SPATIAL_PERC]	COMF, SPATIAL_PERC
Li et al. (2019)	SLC, ATTN_SHIFT, HEC, SPATIAL_PERC	N/A
Pepe et al. (2019)	SURG	N/A
Wu et al. (2018)	ATTN_SHIFT	N/A
Liebert et al. (2016)	ATTN_SHIFT	N/A
Gnanasegaram et al. (2020)	ENG_MOT	ENG_MOT, SPATIAL_PERC
Sun et al. (2020b)	EXP_OUTCOME	EASE_HCI
Park et al. (2020)	ATTN_SHIFT, SPATIAL_PERC	SPATIAL_PERC
Mendes et al. (2020)	SLC	USEF, EASE_HCI, FRUS
Laguna et al. (2020)	SPATIAL_PERC, CONF	SPATIAL_PERC
Dallas-Orr et al. (2020)	N/A	SPATIAL_PERC
Zafar and Zachar (2020)	SPATIAL_PERC, CONF	EASE_HCI, COMF, USEF
Fitski et al. (2020)	DPPC, SPATIAL_PERC	USEF, CONF
Schoeb et al. (2020)	N/A	EASE_HCI, CONF, SLC
Luzon et al. (2020)	N/A	CONF, SPATIAL_PERC
Matsukawa and Yato (2020)	[ATTN_SHIFT → SURG, INC]	N/A
Yang et al. (2020)	SURG, SPATIAL_PERC	SURG
Li et al. (2020b)	[HEC → SURG]	FAT, INC, COMF
Kumar et al. (2020)	SPATIAL_PERC, MM	DPPC, COMF
Li et al. (2020a)	SPATIAL_PERC, SURG	N/A
Gibby et al. (2020)	MM	EASE_HCI
Gu et al. (2020)	ATTN_SHIFT, SURG	N/A
Galati et al. (2020)	ATTN_SHIFT, SURG, MM, SLC	COMF, STRESS, EASE_HCI, DPPC
Viehöfer et al. (2020)	SLC, EXP_OUTCOME, SURG	EXP_OUTCOME
Dennler et al. (2020)	SLC, EXP_OUTCOME, SURG, ATTN_SHIFT	N/A
Kriechling et al. (2020)	SURG, EXP_OUTCOME	N/A
Zorzal et al. (2020)	[ATTN_SHIFT → HEC], SLC, COMF, FAT	DPPC, COMF, EASE_HCI, USEF
Cartucho et al. (2020)	N/A	EASE_HCI, USEF, VIS_OPT, COMF
Rojas-Muñoz et al. (2020b)	[ATTN_SHIFT → MM, SURG]	EASE_HCI, FRUS
Scherl et al. (2020)	N/A	EASE_HCI, COMF
Creighton et al. (2020)	SPATIAL_PERC	N/A
Jiang et al. (2020)	DPPC, MM	N/A
Sun et al. (2020a)	N/A	N/A

Image guidance, where a preoperative segmented imaging model is aligned to the operative view, is the dominating application across several surgical specialities. When providing such guidance and navigation, safety and accuracy becomes crucial. We summarised the achieved accuracy results in Section 8 and noted that there is considerable variation in the reported results, which is related to large variation in terms of conducted experiments and different accuracy measures. Registration can be achieved manually or by identification of point or surface features using an external tracking device. There is no general solution to the problem of registration as yet.

The most common visualisation is of preoperative models. When these are generated from a preoperative scan this requires a segmentation process that must be incorporated into the surgical planning workflow. Medical image segmentation is a huge research area in its own right, with great progress being made. Though this is a vital component of image guidance, we have chosen not include it in this review of OST-HMD AR.

Beyond surgical guidance, other surgical application contexts where accuracy of superimposed holographic content may be less important have been analyzed in this review, such as preoperative planning or surgical training. Due to the variety of surgical contexts, different AR visualisations have been used, such as preoperative models, intraoperative images and intraoperative streaming of video, which all serve different purposes and are rated differently by users in terms of their usefulness.

Phantom experiments dominate, underlining the fact that many such systems are some way from clinical use. Aside from technological limitations, human factors have a major influence on the establishment of OST-HMD assisted applications in the operating room. Attention shift between the surgical site and an external computer monitor is the dominating human factor researchers aim to solve with OST-HMD solutions. These devices lead to other human factor issues, however, such as impaired hand-eye coordination and increased cognitive load that may increase rather than decrease the risk of surgical errors.

### 10.1 Human factor classification

The presented human factors of this review reflect our attempt to identify human user’s individual HCI characteristics in context of OST-HMD assisted surgery, providing an overview of perceptual and HCI related human characteristics that may impact the utility of a proposed novel AR-assisted system. Given that OST-HMD based surgical applications have not replaced respective conventional state of the art methods yet, we feel there is a need to increase awareness of all aspects that may influence the end user’s acceptance of a novel technology being introduced in the operating room. Despite addressing several human factors, OST-HMD based solutions also expose the user to new human factors that may hinder an acceptance of this novel technology in the operating room.

The dominating persistent human factor is the perceived degree of ease and intuitiveness of HCI. These new HCI possibilities may reveal individual performance differences and user preferences even more than conventional computer assisted surgical methods. Overall, it appears that the combination of OST-HMD device, surgical speciality, surgical application context, surgical procedure, proposed AR visualisation and conducted experiments triggers different individual human HCI responses that lead to variation in individual perceived utility. Some attempts have been made to provide standardised analysis in image guidance applications. Zuo et al. (2020) proposed a novel multi-indicator evaluation model for mixed reality surgical navigation systems that evaluate the user’s perception in regards to safety, comfort and efficiency and combines subjective and objective evaluation criteria. Doswell and Skinner (2014) identified the need for HMD-based, scientifically grounded methods that identify HCI related interaction modalities to be addressed to optimise user performance and cognitive load. These comprise information presentation, user input and system feedback. the suggest that an ideal HCI system should be able to adapt these interaction modalities in real-time and in response to the given task as well as environmental and user psychophysiological states.

A taxonomy for mixed reality visualisation in image guided surgery has been proposed by Kersten-Oertel et al. (2012) aiming to introduce a new common framework that facilitates the establishment of validation criteria and should lead to more mixed reality systems being used in daily surgical routine. The paper is well cited and the comprehensive literature review of AR in laparoscopic surgery from Bernhardt et al. (2017) categorises articles according to their taxonomy. But the translation into commercial applications that are used on a daily basis in operating rooms has not materialised as yet. A similar taxonomy tailored to OST-HMD AR would be desirable but is hard to achieve given the widely varying needs of the implementations presented in this review.

### 10.2 Potential machine learning applications

Given the increasing trend of machine learning (ML) applications for medical image processing, such methods are likely to be applied to OST-HMD solutions. However, none of the selected 91 articles contained such a ML application. OST-HMD systems provide a 3D world with a wealth of data, including video images, gesture-based interaction data, eye tracking and generated surface meshes. These could provide rich training data for ML algorithms.

ML algorithms have been proposed for surgical mixed reality applications. Azimi et al. (2018) presented an interactive training and operation ecosystem for mixed reality related surgical tasks that includes data collection for potential ML algorithms. Their system records data from multiple users, such as gaze tracking to indicate which locations in 3D space a surgeon is paying attention to. ML algorithms could then use this data to identify novice surgeons and activate guidance support.

Another example aiming to expand the user’s hands-free interaction possibilities when wearing a HMD was proposed by Chen et al. (2019). Using a self-made HMD with eye-tracking cameras, the authors proposed a deep convolutional neural network to classify gaze trajectories and gaze trajectory gestures. The classified gestures in turn can then trigger different HCI operations. The HoloLens 2 comes with built-in gaze tracking and offers new HCI possibilities that still need to be explored, especially in a surgical setup. A fundamental step in terms of accelerating ML research in the surgical field will be the creation of data bases with relevant user data, which can then serve as inputs for ML algorithms.

### 10.3 Conclusions

The field of OST-HMD assisted surgery has shown a significant recent upward trend in the number of publications as well as the diversity of surgical applications that could benefit from this technology. The release of the Microsoft HoloLens has boosted research into mixed reality surgical applications from 2017 onwards (see table 2). However, comparatively few systems have been used clinically to date and demonstration of utility is rare.

It is worth noting that Dilley et al. (2019), in a screen-based simulation system, compared direct AR with nearby unregistered guidance. Overlaid AR was found to cause inattention blindness, where the augmented view distracts from important events in the real view. This problem arises even when registration is perfect. One option is that guidance information could be presented near to, but not overlaid directly on the surgical view. Such a side-by-side visualisation would allow correctly oriented, but not fully registered model data to be readily available without obscuring or confusing the real view.

The training aspect of OST-HMD visualisation should not be underestimated. The ability to view 3D anatomy and pathology in situ may improve spatial understanding in novice surgeons and reduce the learning curve. Louis et al. (2020) demonstrated improved learning with AR under high fidelity conditions. There is a case for similar experiments to be conducted using OST-HMD AR to provide evidence of proven benefit to learning with these devices.

One potential direction for research is a human factors approach that starts by identifying explicit points or moments in a procedure that may affect patient outcome and then tailors visualisations to improve performance and decision-making for these specific tasks. Demonstration of improved performance on similar specific tasks in the laboratory setting might also lead to a better understanding of the optimal role of AR.

Increasing exposure to AR devices may also improve acceptability of such technology. Taking both technological and human factors into consideration from the outset should lead research towards effective clinical implementations that finally realise the full potential of surgical AR.

## CRediT authorship contribution statement

**Manuel Birlo:** Conceptualization, Methodology, Writing – original draft. **P.J. Eddie Edwards:** Writing – review & editing, Supervision. **Matthew Clarkson:** Supervision, Writing – review & editing. **Danail Stoyanov:** Supervision, Writing – review & editing, Funding acquisition.

## Declaration of Competing Interest

All authors have participated in (a) conception and design, or analysis and interpretation of the data; (b) drafting the article or revising it critically for important intellectual content; and (c) approval of the final version.

This manuscript has not been submitted to, nor is under review at, another journal or other publishing venue.

The authors have no affiliation with any organization with a direct or indirect financial interest in the subject matter discussed in the manuscript

## Figures and Tables

**Table A.1:** Description of AR visualization, Conducted experiments and accuracy of final 91 articles used for quantitative synthesis. Acronyms: PM: Preoperative model; II: Intraoperative image. PI: Preoperative image. IM: Intraoperative model. IV: Intraoperative live streaming video. PV: Preoperatively recorded video. DOC: Documents. COMM: 2D plane with video communication software application (google hangouts etc.). IND: Intraoperative numerical data. SSE: System setup experiment without phanton, cadaver or patient involvement (may contain additional hardware). PE: Phantom experiment. HCE: Human cadaver experiment. AE: Animal experiment. ACE: Animal cadaver experiment. SE: Simulator experiment. SCE: Simulated clinical environment experiment. PS: Patient case study. Abbreviations: Quan: Quantitative study. Qual: Qualitative study.

Study	AR visualizations	Experiments	Reported Accuracy
Chen et al. (2015)	PM: optimal bone drill trajectory, organs, bone structures	Quan: PE: 1.) registration accuracy, 2.) surgical navigation. HCE: 3.) joint screw implantation	1.) 0.809±0.05mm, $1.038^{\circ }\pm 0.05^{\circ }$.
Wang et al. (2016)	PM: 3D pelvis model incl. vessels, optimal bone drill trajectory	Quan: HCE: joint screw implantation	2.7±1.2mm, 3.7±1.1mm, $2.9^{\circ }\pm 1.1^{\circ }$
Deib et al. (2018)	II: radiographic images	Quan: & Qual: PE: Percutaneous vertebroplasty, kyphoplasty and discectomy interventions	N/A
Andress et al. (2018)	II: 2D X-ray images inkl. annotations, IM: guiding lines, planes & spheres, C-arm source position (cylinder)	Qual: SSE: 1.) Calibration, 2.) HMD tracking, 3.)Landmark identification, PE: 4.) K-wire guidance, 5.) Entry point localization (implantation of nails)	1.) 21.4±11.4mm, 2.) 16.2±9.5mm, 3.) 8.76−11.7±(3.21−4.03)mm, 4.) 4.47±2.91 $ 9.84±3.97, 5.) 5.2 mm
Condino et al. (2018)	PM: Anatomical 3D models (incl. bones & muscles), virtual menu with toggle buttons, preoperative plan. IM: optimal tool trajectory	Quan: 1.) System accuracy estimation (perceived AR target positions). Qual: 2.) Subjective workload assessments (NASA Task Load Index)	1.) 0.6 mm
Stewart and Billinghurst (2016)	IM: pose of surgical tool (stack of cyan rings), navigation target (circle)	Quan: PE: 1.) tracked tool positioning & orienting, Qual: 2.) questionnaire	1.) 0.40± 0.78 mm, 2.07 ±$1.68^{\circ }$
Gibby et al. (2019)	PM: virtual trajectories (pedicle screw guidance), lumbar spine 2D & 3D CT images	Quan: PE: 1.) Registration accuracy verification, 2.) Percutaneous placement	1.) 12.99 mm (12.3113.61 mm), 2.) 12.99 mm (12.3113.61 mm), 15.59 mm (12.0218.69 mm)
de Oliveira et al. (2019)	PM: 3D organs incl. fiducial or anatomical markers	Quan: 1.) reliability assessment of virtual-physical mappings, Quan: & Qual: 2.) assessment of superimposed holograms in physical space	1.) 19.74±2.38mm (x), 76.82±3.83mm (y), 2.74±1.96mm (z), $19.74\pm 2.38^{\circ }$, 76.82±3.83, $2.74^{\circ }\pm 1.96^{\circ }$, 2.) 3.2±1.6 mm (RMSE)
Aaskov et al. (2019)	PI: anteroposterior lumbar X-ray 2D images	Qual: PS: 1.) Accuracy and 2.) repeatability validation	1.) 8.77 mm
Liebmann et al. (2019)	PM: targeted screw trajectory, drill entry points. IM: drill angle between current and targeted screw trajectory, 3D trajectory deviation triangle	Quan: PE: guiding wire placement for pedicle screw	2.77±1.46 mm, $3.38^{\circ }\pm 1.73^{\circ }$
Fotouhi et al. (2019a)	II: interventional X-ray images, IM: view frustrum	Quan: SSE: 1.) Hand-eye calibration experiment, PE: 2.) internal fixation of pelvic ring fractures & percutaneous vertebroplasty	1.) 0.43 mm ± 0.34 mm, $0.43^{\circ }$±$0.34^{\circ }$
Lin et al. (2018)	IM: needle visualizations (needle position, orientation & shape, tangential ray)	Quan & Qual: PE: needle insertion task	8.15 mm ± 0.4 mm, 6.54 mm ± 0.294 mm, 6.03 mm ± 0.291 mm
Meulstee et al. (2019)	PM: 3D objects (cube)	Quan: PE: 1.) Tight-Fit & Loose-Fit Accuracy Evaluation	1.) 0.7 ± 0.2 mm, 2.) 2.3 ± 0.5 mm
Guo et al. (2019)	3D calibration cubes	Quan: SSE: calibration accuracy	below 6 mm, up to $5^{\circ }$
Brun et al. (2019)	PM: 3D heart models	Quan: SSE: patient based heart model analysis (anatomy identification & diagnosis)	N/A
Li et al. (2017)	PM: 3D coronay arteries models	Quan: SSE: Dynamic and static gesture recognition	N/A
Zou et al. (2017)	PM: 3D cardio artery vascular models	Quan: SSE: hand gesture recognition rate validation	N/A
Liu et al. (2019)	PM: 3D heart, spine & cathether models, 3D catheter path planning	Quan: PE: catheter navigation under C-arm fluoroscopy guidance	0.425±0.021 mm (registration), 0.29±0.19 mm (catheter position)
Kaneko et al. (2016)	II: ultrasound images	Quan: SE: sonographic guided jugular vein catheterization	N/A
Kuhlemann et al. (2017)	PM: 3D patient surface mesh, vascular tree, catheter position, registration landmarks, canvasses (1.) 2D CT slide, 2.) catheter point of view perspective inside vascular tree)	Quan: PE: 1.) calibration, 2.) catheter insertion & navigation, 3.) Likert scale questionnaire evaluation	1.) 1.) 4.34±0.709 mm (RMSE point-to-point correspondence)
Karmonik et al. (2018)	PM: complex medical vascular & blood flow 3D image data	Quan: SSE: Evaluation of vascular & blood flow image data	N/A
Frantz et al. (2018)	PM: 3D skull visualizations, localization markers	Quan: PE: 1.) Manual registration, 2.) Maintaining hologram registration via continuous camera tracking	1.) 4.39 ± 1.29 mm, 2.) 1.4 ± 0.67 mm (mean perceived drift)
Yoon et al. (2017)	II: 2D neuronavigation images	Quan: PS: pedicle screw placement	N/A
Pietruski et al. (2019)	II: 2D navigation monitor. PM: 3D mandible model, 3D osteotomy cutting guides (planes) & navigated surgical saw, IND: cutting guide deviation coordinate system	Quan: PE: osteotomies: 1.) augmented navigation system monitor & 2.) superimposition of surgical plan	1.) 1.79 ± 0.94 mm, 3.67 ±$3.67^{\circ }$, 2.) 2.41 ± 1.34 mm, 7.14 ±$5.19^{\circ }$
Mitsuno et al. (2017)	PM: Preoperative & ideal postoperative 3D facial surface and facial bones	Quan: PS: reconstructive surgeries (facial fractures or deformities)	30−40 mm (display error)
Pratt et al. (2018)	PM: 3D bony, vascular, skin & soft tissue structures, vascular perforators, bounding box	Quan: PS: flap surgery	N/A
Fotouhi et al. (2020)	PM: 3D virtual robot arm, 2D reflective AR display	Quan: PE: Registration with 1.) and without 2.) reflective AR displays, 2.) Simulated robot-assisted trocar placement	1.) 16.5±11.0 mm, 2.) 30.2±23.9 mm (misalignment error)
Qian et al. (2018)	PM: & II: 3D plane with endoscopy visualization, IM: viewing frustrum, PM: endoscope, robotic & hand-held instruments	Quan: SSE: 1.) Display calibration, 2.) Camera calibration. PE: Visualization performance evaluation	1.) 4.27±3.09 mm
Song et al. (2018)	PI: 2D radiographic images with guidance information, IM: 3D drill guidance information	Quan: PE: 1.) Accuracy evaluation, 2.) Tool navigation & guidance	1.) Avg: 0.46 mm, Max: 0.86 mm, Avg: $1.17^{\circ }$, Max: $2.10^{\circ }$
Hiranaka et al. (2017)	II: fluoroscopic video	Quan: PE: Guide wire insertion into femur	2.6±0.02 mm
Katić et al. (2015)	PM: & IM: position, depth & alignment of planed & actual dental drill, injury avoidance warnings, drill heads	Quan: 1.) SSE: Calibration accuracy, 2.) ACE: Implant placement	1.) 3.01±3.01 mm, 2.) <2.5 mm (implant deviation),
Borgmann et al. (2016)	II: preoperative CT scan	Quan: PS: different urological procedures, Likert scale questionnaire	N/A
Unberath et al. (2018)	IM: Live 3D point cloud (C-arm pose)	Quan: PE: pelvic trauma surgery	51.6±19.2 mm, $1.54\pm 0.92^{\circ }$
Sauer et al. (2017)	PM: 3D hepatic artery, portal vein, hepatic veins, liver tumor, liver capsule	Quan: PS: open hepatic surgery	N/A
Armstrong et al. (2014)	COMM: google hangouts, DOC: articles from senior author	Quan: PS: reconstructive limb salvage procedure	N/A
Mahmood et al. (2018)	PM: 3D anatomical models, 3D ultrasound streaming plane	Quan: SE: transesophageal echocardiography	N/A
Rojas-Muñoz et al. (2019)	PM: 3D graphical annotations (incision lines, surgical instruments)	Quan: SE: 1.) anatomical marker placement, 2.) mock abdominal incision	1.) 11.37±0.72 mm
Li et al. (2019)	PM: 3D liver structure (intraoperatively updated), tumor, virtual needle, registration landmarks	Quan: 1.) PE: Registration accuracy validation, 2.) AE: needle insertion operation	1.) 2.24 mm (avg. target registration error)
Rojas-Muñoz et al. (2020a)	PM: 3D graphical annotations (lines & models)	Quan: HCE: leg fasciotomy	N/A.
Pelanis et al. (2020)	PM: 3D liver incl. parenchyma, portal, hepatic veins & lesion	Quan: SSE: Identification of liver segments	N/A
Pepe et al. (2019)	PM: 3D landmarks, 3D tumors, 3D axial facial CT slice	Quan: PE: Automatic registration after user calibration	x: 3.3±2.3 mm y: -4.5±2.9 mm z: -9.3±6.1 mm
Nguyen et al. (2020)	PM: 3D patient head incl. skin, skull & spine	Quan: PE: Registration accuracy. 3 registration methods: 1.) Keyboard, 2.) Tap to Place, 3.) 3-Point correspondence matching	1.) X Axis: $5\pm 5^{\circ }$ Y Axis: -$5.9\pm 5.9^{\circ }$ Z Axis: $6.8\pm 5.9^{\circ }$; displacement: XY Plane: 2.9±1.8 mm ZY Plane: 1.8±1.2 mm XZ Plane: 1.6±0.9 mm
Zhou et al. (2019b)	PM: 3D organs, needle (actual & preoperative plan)	Quan: 1.) PE: & 2.) AE: needle insertion	1.) 0.664 mm, $4.74^{\circ }$, 2.) 1.617 mm, $5.574^{\circ }$
Pietruski et al. (2020)	PM: 3D bones, surgical plan: control points, osteotomy trajectories, navigated saw, 2D digital coordinate system	Quan: PE: osteotomy	4.1±2.29 mm, $5.08\pm 3.64^{\circ }$, $4.97\pm 2.91^{\circ }$
Chien et al. (2019)	PM: 3D patient skin surface	Quan: PE: Alignment (different data sparsity percentages are tested but we refer only to 100 % of floating data being used)	5 reference points alignment error RMSE: Avg.: 0.932 mm, Min: 0.37 mm, Max: 1.49 mm
Zhang et al. (2019)	PM: 3D intracranial structure, lesion	Quan: PS: Craniotomy	N/A.
Heinrich et al. (2019)	PM: 3D Needle insertion guidance visualization options: 1.) planes, 2.) lines, 3.) cone rings	Quan: PE: 1.) Registration accuracy estimation: a) Angle measurement of displayed lines I, b) Angle measurement of displayed lines II, c) Tracked normal vector accuracy, d) Tracked normal vector accuracy, 2.) Comparison study	1 a.) $0.76\pm 0.11^{\circ }$, 1 b.) frontal viewing pos. $1.90\pm 1.82^{\circ }$, $45^{\circ }$ viewing pos. $4.28\pm 4.09^{\circ }$, lateral viewing pos. $7.94\pm 7.75^{\circ }$, 1 c.) $0.72\pm 0.41^{\circ }$, 1 d.) X $ Y marker: $0.27\pm 0.21^{\circ }$, X $ Z marker: $0.31\pm 0.22^{\circ }$, Y $ Z marker: $0.38\pm 0.36^{\circ }$
Zhou et al. (2020)	3D anatomy (skin, bones, tumor tissue), virtual needles (planning & detected), seeds, 2D control panel	Quan: 1.) PE: & 2.) AE: brachytherapy of tumors	Avg. needle location error: 1.) 0.957 mm, 2.) 2.416 mm
Wellens et al. (2019)	PM: 3D kidneys incl. tumor, arteries, veins, urinary collecting structures	Quan: SSE: Assessment of anatomical structures	N/A
Fotouhi et al. (2019b)	IM: 3D anatomical structures, C-arm principle axis	Quan: SSE: 1.) calibration accuracy, PE: 2.) Target augmentation error, 3.) Augmented surgical visualization	1.) 5.7±0.26 mm, 2.) 10.8±3.45 mm
Baum et al. (2020)	PM: 3D patient skin surface, brain, intra-cortical lesion	Quan: PE: Target Localization	N/A
Liounakos et al. (2020)	II: live endoscopic camera image	Quan: PS: Lumbar discectomy	N/A
Jalaliniya et al. (2017)	COMM: videoconferencing application, PI: patient records	Quan: PE: SCE: Mobile access to patient records, telepresence	N/A
Rynio et al. (2019)	PM: 3D arterial system, aneurysm, bones, PI: 2D image with volume rendering, arterial diameters & planning notes	Quan: PS: Abdominal aortic aneurysm repair	N/A
Boillat et al. (2019)	PM: 2D surgical safety checklist	Quan: SSE: time-out checklist execution	N/A
Zhou et al. (2019a)	PM: 3D tooth, cone (endoscope view frustrum), probe alignment cyclinder & planes, II: 2D imaging	Quan: PE: 1.) Augmentation quality evaluation, 2.) Dental decay localization	31±11 px (keypoint displacement)
Schlosser et al. (2019)	IND: 2D screen incl. patient heart rate, blood pressure, blood oxygen saturation, alarm notifications	Quan: & Qual: PS: vital sign monitoring, Quan: situation awareness measurement	N/A
Ponce et al. (2014)	IV: hybrid image (surgical field combined with hands of remote surgeon)	Quan: PS: shoulder replacement	N/A
Dickey et al. (2016)	IV: interactive video display incl. cursor moved by supervising physician, PV: training guide	Qual: & Quan: SSE: user survey	N/A
Al Janabi et al. (2020)	PI: CT images, IV: live fluoroscopy, endoscopic view	Qual: & Quan: SE: mid-ureteric stone removal	N/A
Wu et al. (2018)	PM: 3D patient anatomy (e.g. head, intracranial vascular tissue)	Quan: PE: dummy head alignment test	<3 mm (Avg. Target Registration Error)
El-Hariri et al. (2018)	PM: 3D bone structures, fiducial markers	Quan: PE: accuracy assessment	Fiducual marker comparisons (RMSE): x: 3.22 mm, y: 22.46 mm, z: 28.30 mm
Liebert et al. (2016)	IND: 2D screen incl. patient arterial line blood pressure, heart rate, heart rhythm, pulse oximetry, respiratory rate	Quan: SE: Vital signs monitoring during bronchoscopy	N/A
Gnanasegaram et al. (2020)	PM: 3D ear anatomy	Quan: SSE: spatial exploration of holographic ear model	N/A
Sun et al. (2020b)	PM: 3D catheter	Quan: SSE: 1.) Stability measurement of tracking algorithm, 2.) testing of tracking accuracy 3.) latency test using third-party tracker, 4.) HCE: EDV performed on a cadaveric head	2.) avg. distance from catheter tip to corresponding grid intersections (2D plane): 0.58 mm, overall avg. accuracy on all 3 grid faces: 0.85 mm (3D space)
Park et al. (2020)	PM: 3D volumes from MRI images	Quan: AE: transarterial embolization of hepatocellular carcinoma (HCC)	N/A
Mendes et al. (2020)	PM: 3D model of body simulator’s external surface (upper torso) and 3D vascular structures	Quan: SE: tracked needle insertion	N/A
Laguna et al. (2020)	PM: 3D elbow fractures (bones)	Quan: SSE: Orthopedic surgeons’ assessment of 3D AR models for presurgical planning in complex pediatric elbow fractures	N/A
Dallas-Orr et al. (2020)	PM: 3D spine model with a vascular model overlay	Quan: SSE: 3D model measurement using circumference and angle tools of standard-of-care PACS software	N/A
Zafar and Zachar (2020)	PM: 3D human skull	Quan: SSE: digital anatomy session with the HoloHuman virtual anatomy training software	N/A
Fitski et al. (2020)	PM: 3D intraparenchymal arteries and veins, kidney, tumor	Quan: PS: Preoperative planning of patients eligible for nephron-sparing surgery (NSS)	N/A
Schoeb et al. (2020)	PV: 2D plane with catheter placement instruction guidance	Quan: SE: Bladder catheter placement using a male catheterization-training model	N/A
Luzon et al. (2020)	PM: 3D anatomy models (e.g. vascular model)	Quan: PE: registration and needle placement	Target error distance: x-axis: 2.9757±1.33396 mm, y-axis: 2.2790±1.44992 mm, z-axis: 2.7844±.91323mm
Matsukawa and Yato (2020)	II: fluoroscopic 2D image	Quan: PS: single-segment posterior lumbar interbody fusion (PLIF) at L5S1	N/A
Yang et al. (2020)	PM: 3D portal vein and hepatic vein, liver	Quan: 1.) AE: dogs: simulated percutaneous puncture of the portal vein and simulated TIPS, 2.) PE: liver phantom experiment	N/A
Li et al. (2020b)	PM: 3D internal organs, PI: CT images, IM: progress view of the virtual planned target, needle path, skin entry point and needle end	Quan: PE: 1.) image overlay accuracy using 3D abdominal phantom, 2.) needle placement performance using tissue phantom	1.) Total target overlay error over 336 targets: 1.74±0.86 mm. Needle overlay angle: $0.41\pm 0.23^{\circ }$
Kumar et al. (2020)	PM: 3D liver and heart, slicing tool (plane)	SSE: visualisation of patient-specific models and hologram interaction (rotate, scale and move)	N/A
Li et al. (2020a)	PM: 3D target organs and tumors (kidney, tumor, renal vessels, renal collection system, skin, skeleton, liver, spleen), PI: MR results, IV: laparoscopic video stream	Quan: PS: Prospective review of patients with stage T1N0M0 renal tumors who untervent laparoscopic partial ephrectomy	N/A
Gibby et al. (2020)	PI: 3D plane with axial CT image, PM: needle trajectories in correct spatial orientation over patient	Quan: 1.) PE: control data experiment (needle navigation) using skull with ballistic gelatin and radiopaque balls (targets), 2.) PS: interventional spine procedures	1.) 0.998±1.66 mm (mean error of needle tip to targeted ball). Mean distance from model surface to targeted ball: 79.42±15.33 mm, 2.) Mean error of needle to target: 1.73±2.20 mm
Gu et al. (2020)	PM: 3D glenoid and planed drilling path	Quan: PE: 1.) inside-out registration (via HoloLens depth sensing camera), 2.) accuracy evaluation of inside-out registration using outside-in tracking with optical tracker, 3.) registration with surface digitisation	1.) Inside-out registration accuracy compared with external tracking (optical trackser is used to verify inside-out tracking): translation (max: 21.82±2.33 mm), rotation: max. $8.10\pm 2.89^{\circ }$
Galati et al. (2020)	PM: 3D patient anatomy	Quan: PS: open abdomen surgeries	N/A
Viehöfer et al. (2020)	PM: 3D foot	Quan: PE: distal osteotomy	Mean deviation between osteotomy plane and target plane perpendicular to the second metatarsal (anterior direction): 1.) Experienced surgeons: $4.9\pm 4.2^{\circ }$, 2.) less experienced surgeons: $6.4\pm 3.5^{\circ }$
Dennler et al. (2020)	PM: vertebral body	Quan: PE: drilling pilot holes in lumbar vertebra sawbones models	average minimal distance of the drill axis to the pedicle wall (MAPW): 1.) Expert surgeons: 5.0±1.4 mm, novice surgeons: 4.2±1.8 mm
Kriechling et al. (2020)	PM: planned drill trajectory, IM: current drill trajectory, deviation in degrees and millimeters	Quan: PE: Using 3D printed scapula based on scans of human cadavers: Guidewire positioning of the central back of he	mean deviation of placed guidewires from the planned trajectory: $2.7\pm 1.3^{\circ }$, mean deviation to the planned entry point of the placed guidewires: 2.3±1.1 mm
Zorzal et al. (2020)	IV: 2D plane with laparoscopic video feed, PI: 2D plane with MR image sclices	Qual: & Quan: SE: laparoscopic training simulator incl. MR images and pre-recorded laparoscopic video feed	N/A
Cartucho et al. (2020)	PM: 3D organ models (brain and liver), PI: 2D planes with volumetric MRI/CT imaging data (with scrolling bar), 2D plane with intraoperative data (pCLE, iUS) (with transparency adjustment up and down arrows))	Quan: SSE: interaction with the visualisation components and exploration of holographic functionalities	N/A
Rojas-Muñoz et al. (2020b)	PM: 3D annotations (incision lines) and 3D surgical tools	Quan: SE: performing cricothyroidotomies in a simulated austere scenario (smoke and loud noises of gunshots and explosions)	N/A
Scherl et al. (2020)	PM: 3D mandible, parotid, tumor, head, grey circles, operating menu (buttons), PI: 2D MRI images	Quan: PS: live parotid surgery: study persons who did not participate in the actual surgery performed manual ologram	Manual Registration accuracy using fiduical markers on the head: outer borders of face: 10.09±4.23mm, arotid: 13.39±4.71 mm, Tumor: 13.29±5.66 mm
Creighton et al. (2020)	PM: 3D skull and temporal bone	Quan: PE: Evaluation of manual target registration error using skull model	Target registration error: 10.62±5.90 mm10.62
Jiang et al. (2020)	PM: 3D vascular map, surrounding soft tissues, marker	Quan: PE: Precision verification of the vascular localization system	mean errors (under different conditions): min: 1.35±0.43 mm, max: 3.18±1.32
Sun et al. (2020a)	PM: 3D planned mandibular reconstruction result	Quan: PE: 1.) Accuracy validation experiment for OST-HMD calibration (3D printed skull with fiducials), 2.) Calibration method testing, 3.) PS: mandibular reconstruction	1.) Avg. root-mean-square error of control points between rendered object and skull model: 1.30±0.39
